# Supplementary material for: Directed evolution of a TNA polymerase identifies independent paths to fidelity and catalysis
Source: Nat Commun. 2025 Dec 19;17:925. doi: 10.1038/s41467-025-67652-1 (PMC12830623; doi:10.1038/s41467-025-67652-1)
Supplement: Supplementary file 1 — Supplementary Information [file 41467_2025_67652_MOESM1_ESM.pdf]

Table of Contents

Supplementary Figures

Supplementary Figure 1.	Structural overlay of Kod and 10-92 closed ternary conformations.
Supplementary Figure 2.	Conformational rearrangement of the thumb subdomain.
Supplementary Figure 3.	Sequence homology-based fitness landscape of TNAP variants
Supplementary Figure 4.	Sequence alignment of TNAPs
Supplementary Figure 5.	Workflow for TNAP fidelity measurements in hydrogel particles
Supplementary Figure 6.	TNAP fidelity raw data and statistical analysis
Supplementary Figure 7.	TNAP kinetics and substrate specificity
Supplementary Figure 8.	TNAP thermal challenge
Supplementary Figure 9.	Polymerase domains
Supplementary Figure 10.	Sequence and secondary structure alignment of Kod and 10-92
Supplementary Figure 11.	Structural comparison of the finger subdomain of Kod, Kod-RI, Kod-RSGA and 5-270 in the binary complex
Supplementary Figure 12.	Active site comparison
Supplementary Figure 13.	Synthesis of 2′-deoxy-α-L-threofuranosyl thymine triphosphate
Supplementary Figure 14.	^{31}P NMR spectrum of compound 2
Supplementary Figure 15.	^{31}P NMR spectrum of compound 3
Supplementary Figure 16.	HPLC analysis of compound 3
Supplementary Figure 17.	Multidimensional analysis of the closed ternary complex of TNAP structures based on RMSD
Supplementary Figure 18.	Distance of mutations to active site
Supplementary Figure 19.	AlphaFold3 prediction overview
Supplementary Figure 20.	AlphaFold3 prediction confidence
Supplementary Figure 21.	AlphaFold3 prediction of 5-270 binary structure
Supplementary Figure 22.	Comparing AlphaFold3 predictions to experimental structures
Supplementary Figure 23.	Visualizing AlphaFold3 predictions against experimental structures.
Supplementary Figure 24.	Active site pocket volume
Supplementary Figure 25.	2Fo-Fc electron density maps of the active site
Supplementary Figure 26.	Polder maps of key residues in the active site
Supplementary Figure 27.	Active site geometry comparing two independently solved structures of 5-270 in a closed ternary complex
Supplementary Figure 28.	Catalysis-enhancing mutations mapped over polymerase domain
Supplementary Figure 29.	Polder maps of mutated residues in 5-270, 8-64, and 10-92
Supplementary Figure 30.	Polder maps of mutated residues in DNAP and TNAPs relative to the catalytic aspartate residues

Supplementary Tables

Supplementary Table 1.	DNA oligonucleotides
Supplementary Table 2.	Data collection and refinement statistics for binary structures
Supplementary Table 3.	Data collection and refinement statistics for ternary structures
Supplementary Table 4.	Base pair parameters
Supplementary Table 5.	Integrated mutational analysis

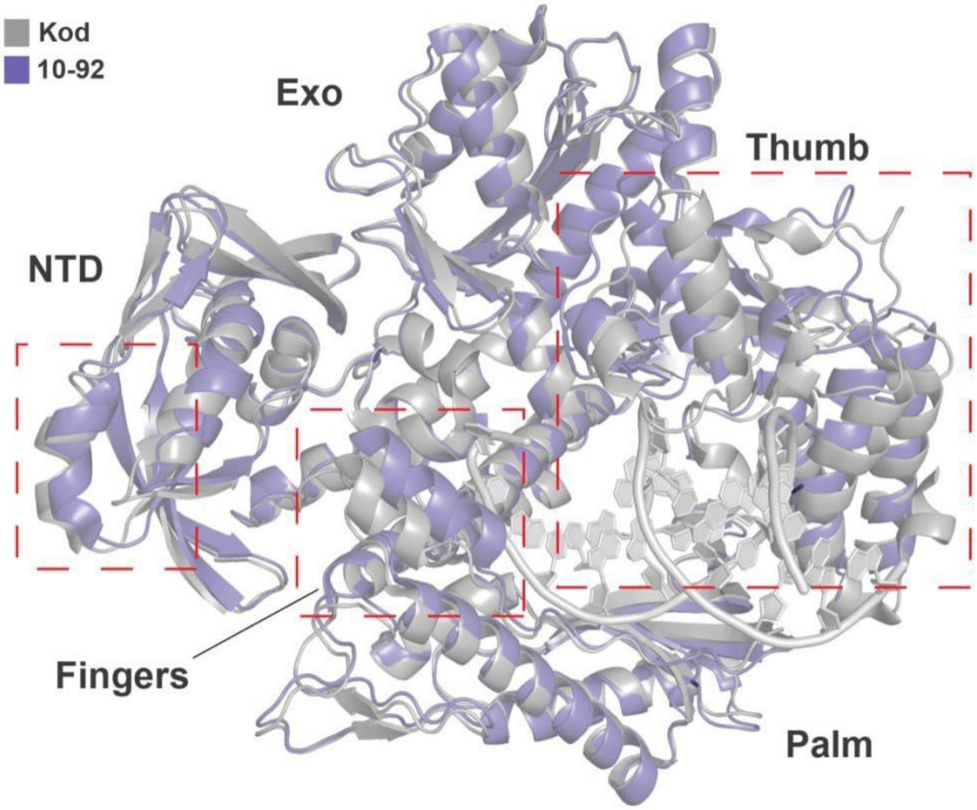

Supplementary Figure 1. **Structural overlay of Kod and 10-92 closed ternary conformations.** Red dashed boxes depict areas of large conformational difference between Kod (transparent grey, PDB: 5OMF) and 10-92 (transparent purple, PDB: 8T3X). For clarity, only the Kod-bound duplex is displayed.

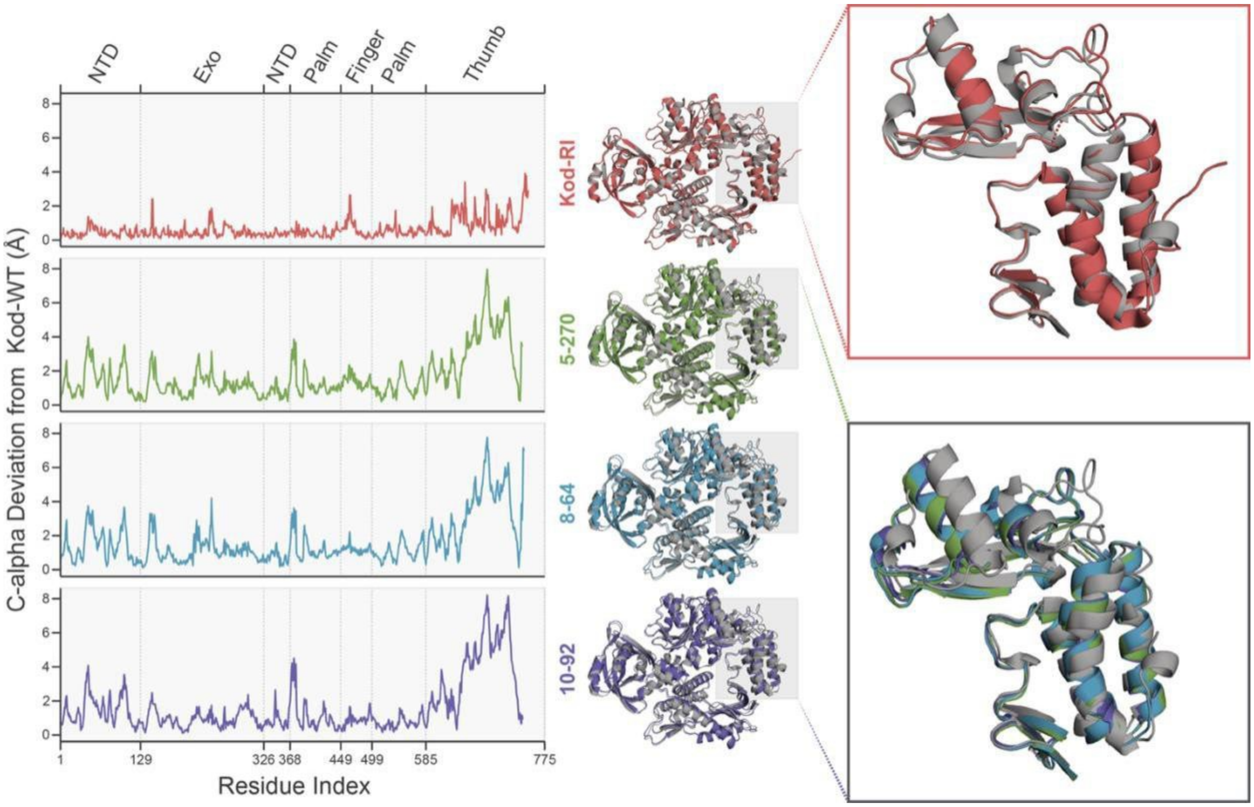

Supplementary Figure 2. **Conformational rearrangement of the thumb subdomain.** Left, the distance in angstroms from all resolved C-α positions in the ternary structures of Kod-RI (red), 5-270 (green), 8-64 (blue), and 10-92 (purple) relative to their position in Kod. Middle, structural overlays of TNAPs aligned to Kod (gray). Right, magnified view of the thumb subdomain showing the structural similarity between Kod-RI and Kod and structural differences between 5-270, 8-64, and 10-92 relative to Kod.

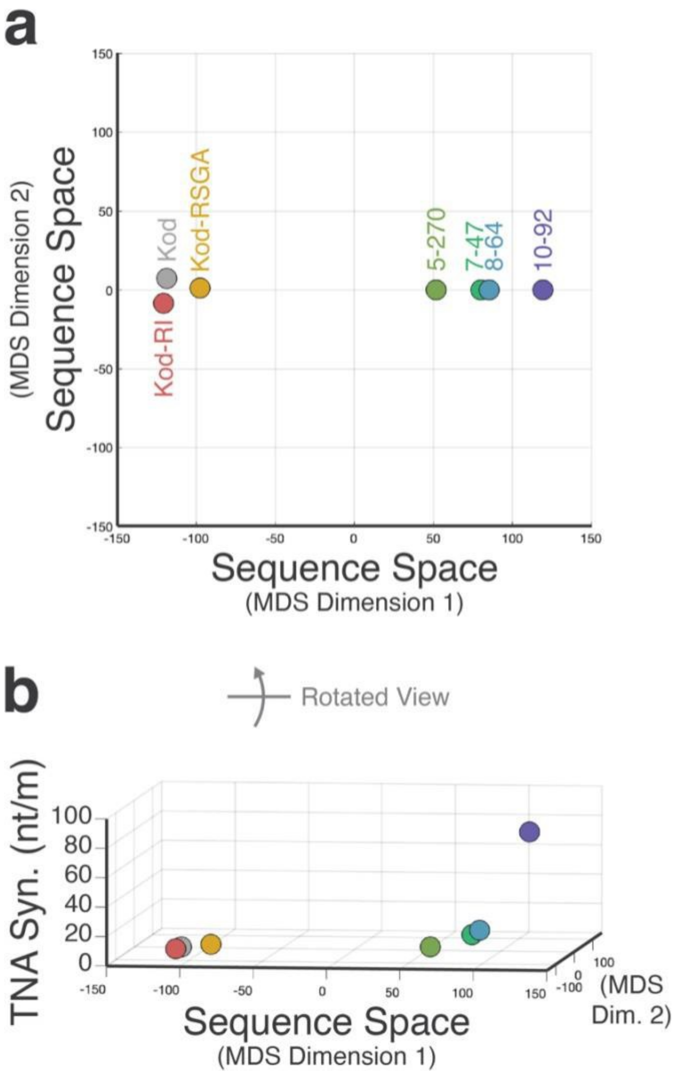

Supplementary Figure 3. Sequence homology-based fitness landscape of TNAP variants. a, Two-dimensional representation of polymerase relationships derived from pairwise sequence alignment scores calculated with the EMBOSS Needle global alignment tool. Pairwise scores were assembled into a distance matrix and analyzed by classical multidimensional scaling (MDS). Each circle represents a polymerase variant, with closer positions indicating higher sequence homology. **b**, Rotated view of the same projection with an additional z-axis corresponding to TNA synthesis activity (nt min^{-1}) as reported in Supplementary Figure 7.

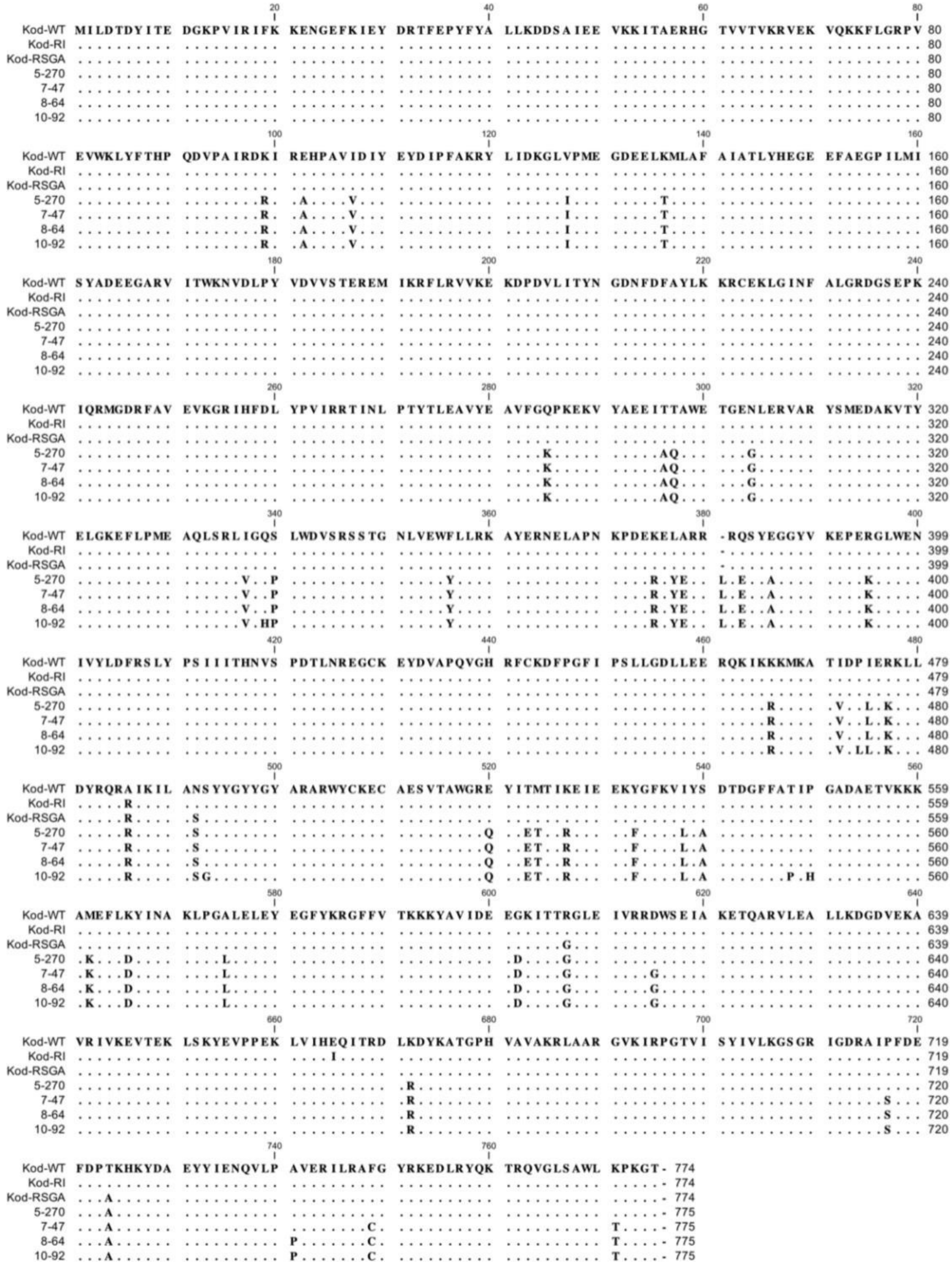

Supplementary Figure 4. Sequence alignment of TNAP generations. Conserved residues are denoted as (.), while mutations are listed as single letter amino acid abbreviations. Alignment was generated in CLC Main Workbench.

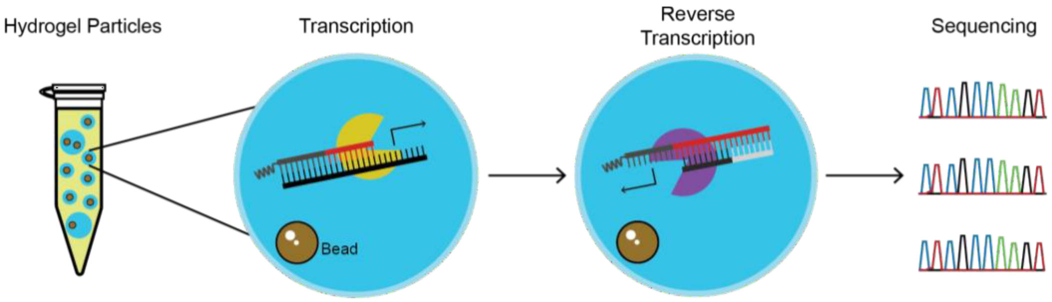

Supplementary Figure 5. **Workflow for TNAP fidelity measurements in hydrogel particles.** A single-stranded DNA template is hybridized to a DNA primer crosslinked to the hydrogel matrix and extended with a TNAP and tNTPs for 1 hour at 55°C. The DNA template is then stripped to display the TNA product strand. The displayed TNA strand is hybridized to an overhang DNA primer and reverse transcribed with Bst DNA polymerase and dNTPs for 4 hours at 50°C. cDNA is amplified by PCR, TOPO cloned, and sequenced. TT mismatches in the primer and template are used to confirm that each sequence passed through a round of TNA replication (DNA→TNA→DNA).

a

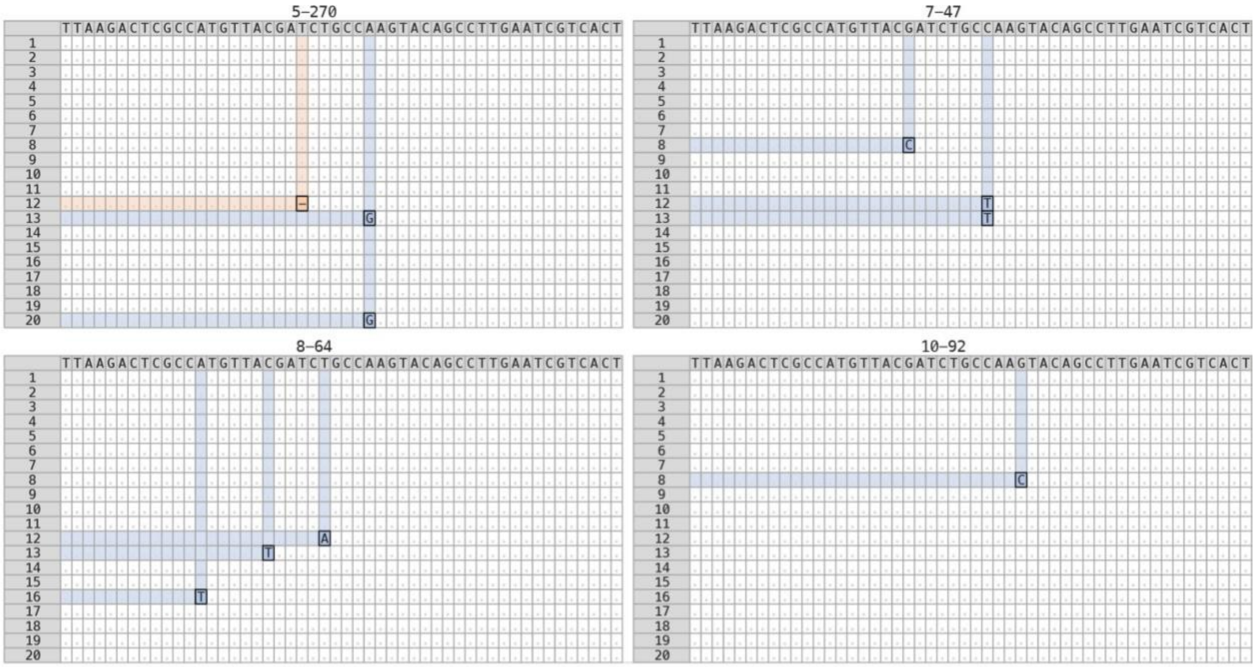

b

Experiment		(Substitution)			(Substitution + In/Del)		
TNAP	Reads (nt)	Errors	Error Rate	Fidelity (%)	Errors	Error Rate	Fidelity (%)
Kod-RSGA	1677	16	0.010	99.046	24	0.014	98.569
5-270	1000	2	0.002	99.800	3	0.003	99.700
7-47	1000	3	0.003	99.700	3	0.003	99.700
8-64	1000	3	0.003	99.700	3	0.003	99.700
10-92	1000	1	0.001	99.900	1	0.001	99.900

Binomial Two-Proportion Z-Test (Substitution)				
Comparison	$\Delta_{Fidelity}$ (pp)	z	p-Value	Statistacal Significance
Kod-RSGA → 5-270	0.754	2.309	0.0209	Significant (p<0.05)
5-270 → 7-47	-0.100	-0.448	0.6543	Not Significant
7-47 → 8-64	0.000	0.000	1.0000	Not Significant
8-64 → 10-92	0.200	1.001	0.3168	Not Significant

Binomial Two-Proportion Z-Test (Substitution + In/Del)				
Comparison	$\Delta_{Fidelity}$ (pp)	z	p-Value	Statistacal Significance
Kod-RSGA → 5-270	1.131	2.833	0.0046	Significant (p<0.05)
5-270 → 7-47	0.000	0.000	1.0000	Not Significant
7-47 → 8-64	0.000	0.000	1.0000	Not Significant
8-64 → 10-92	0.200	1.001	0.3168	Not Significant

Supplementary Figure 6. TNAP fidelity raw data and statistical analysis. (a) Raw Sanger sequencing alignments from a single cycle of TNA replication by the indicated TNAP variants. The expected DNA sequence is shown 5′→3′. Correct base incorporations are marked with (.), and substitutions or insertion/deletion events are outlined in black boxes with guiding blue or orange highlighting. (b) Fidelity values represent the aggregate accuracy determined from at least 1,000 nucleotides per enzyme, calculated both with and without indels. Statistical significance of fidelity changes between successive TNAP generations was assessed using a binomial z-test to show the significance between Kod-RSGA and 5-270.

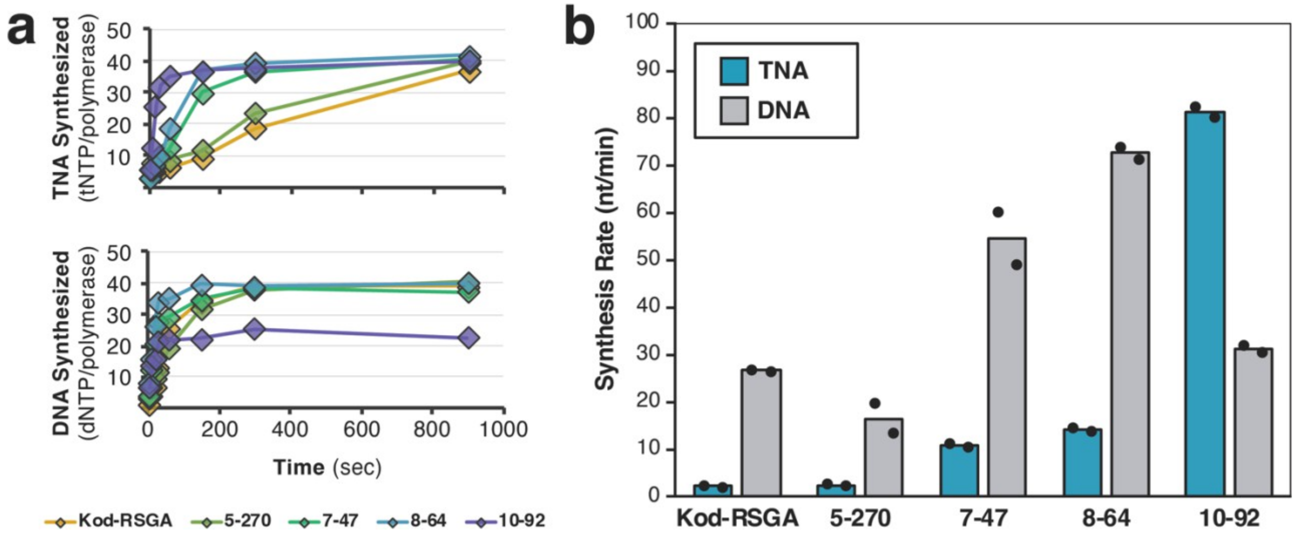

Supplementary Figure 7. TNAP kinetics and substrate specificity. a, Kinetic time-course of primer-extension reactions catalyzed by Kod-RSGA, 5-270, 7-47, 8-64, and 10-92 using TNA (tNTP) or DNA (dNTP) triphosphates, average of n=2. The slope of the linear range corresponds to the rate of synthesis and is plotted in panel **b** as nucleotides per minute.

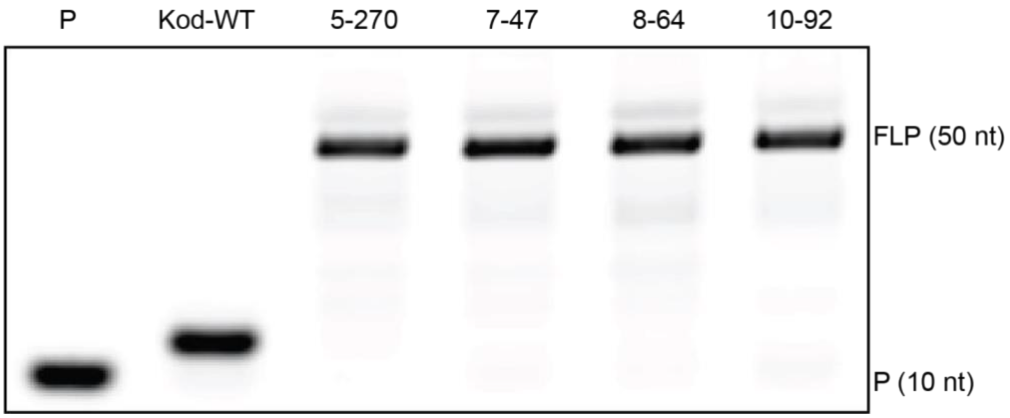

Supplementary Figure 8. TNAP thermal challenge. TNA synthesis activity was assessed after exposing the polymerases to 6 hours of incubation at 90°C. Reactions were performed by incubating a 5' IR680-labelled DNA primer–template duplex (1 μM) with 1 μM polymerase and 100 μM tNTPs in 1x ThermoPol buffer for 30 minutes at 55°C. P, primer; FLP, full-length product.

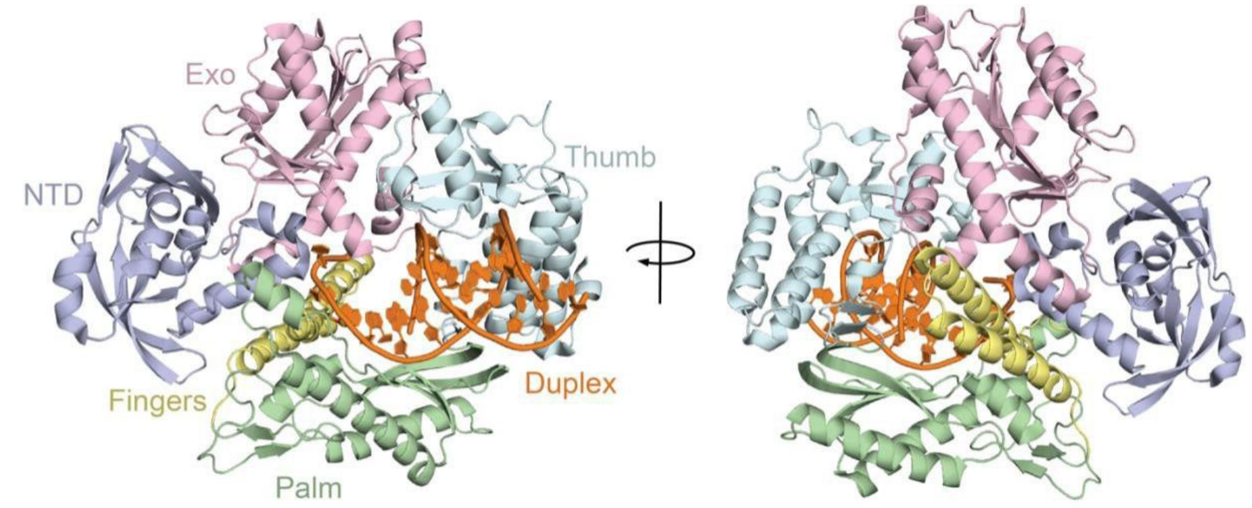

Supplementary Figure 9. Polymerase domains. The architecture of B-family polymerases colored by domain, mapped on the closed ternary structure of wildtype Kod (PDB ID: 5OMF).

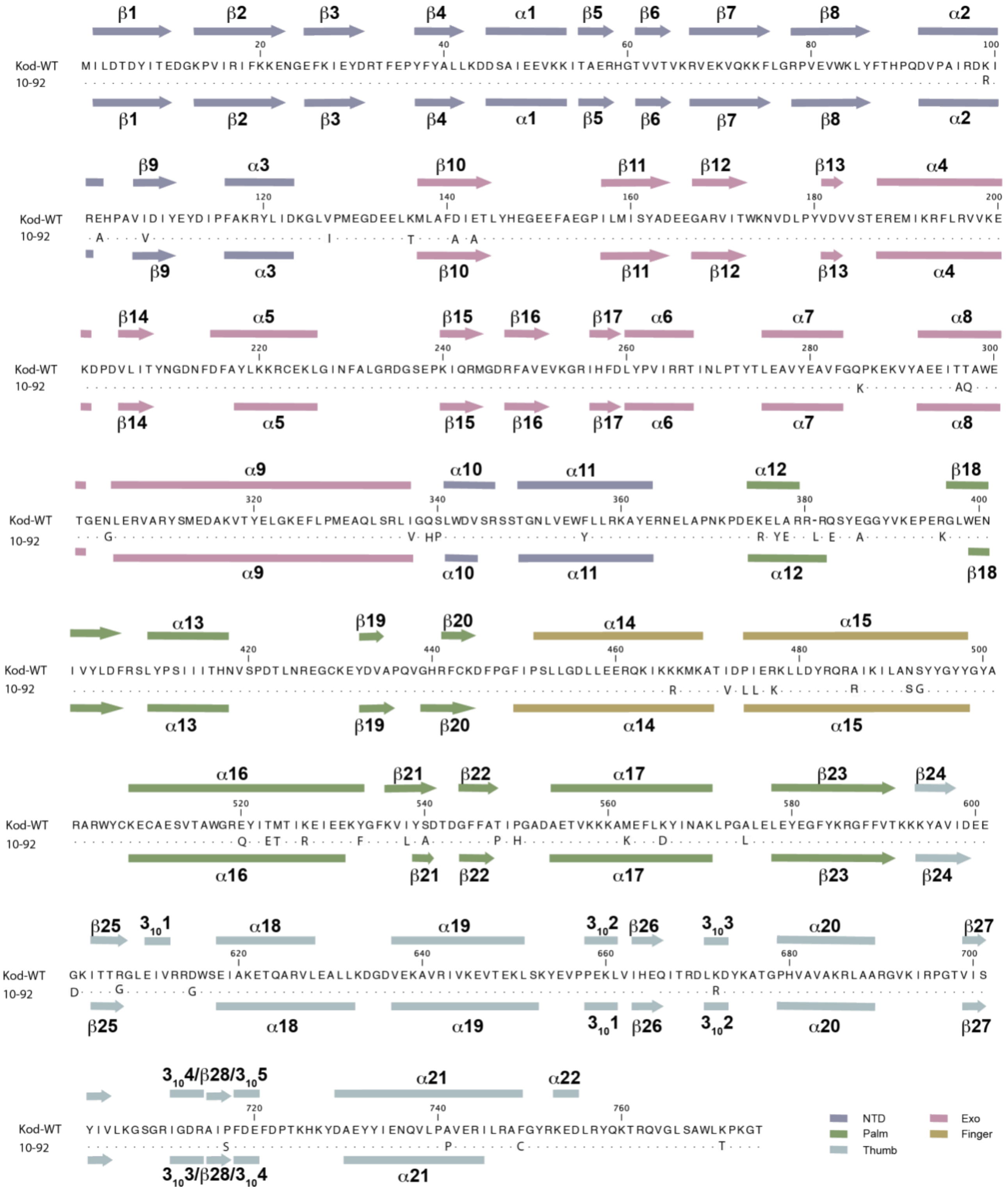

Supplementary Figure 10. Sequence and secondary structure alignment of Kod and 10-92. Conserved residues are denoted as (.), while mutations are listed as single letter amino acid abbreviations. Secondary structures of Kod and 10-92, as determined by DSSP and PyMol, are depicted above and below the sequence alignment, respectively. Secondary structural elements are colored by domains, which are color-matched to Supplementary Figure 9.

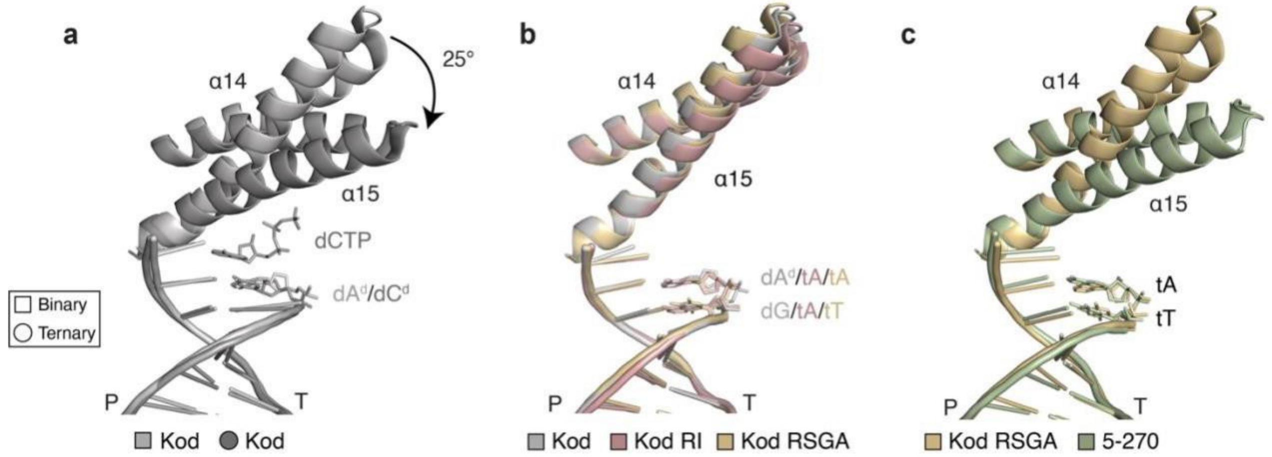

Supplementary Figure 11. Structural comparison of the finger subdomain of Kod, Kod-RI, Kod-RSGA, and 5-270 in the binary complex. **a**, Overlay of the finger subdomains of Kod binary (light gray, PDB: 4K8Z) and closed ternary (dark gray, PDB: 7OMB) complexes. **b**, Overlay of the finger subdomains of Kod binary (light gray), Kod-RI binary (pink, PDB: 5VU9), and Kod-RSGA binary (yellow, PDB: 7RSU) complexes. **c**, Overlay of the finger subdomains of Kod RSGA and 5-270 (green) binary complexes. Structural overlays reveal the binary complex of 5-270 is structurally distinct from the binary complex of Kod, Kod-RI, and Kod-RSGA.

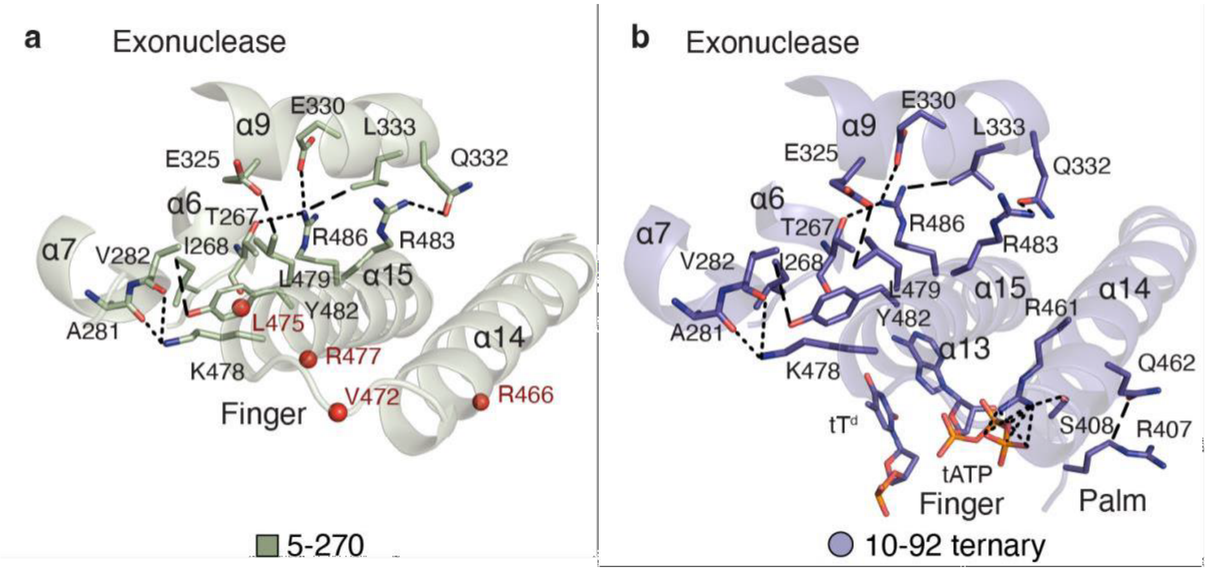

Supplementary Figure 12. Active site comparison. The active site of **a**, the 5-270 binary structure reveals a closed finger conformation similar to **b**, the 10-92 closed ternary structure. Short dashed lines denote electrostatic interactions, long dashed lines show hydrophobic interactions. Residue labels: natural residues found in Kod DNAP (black); acquired mutations (red spheres, labels).

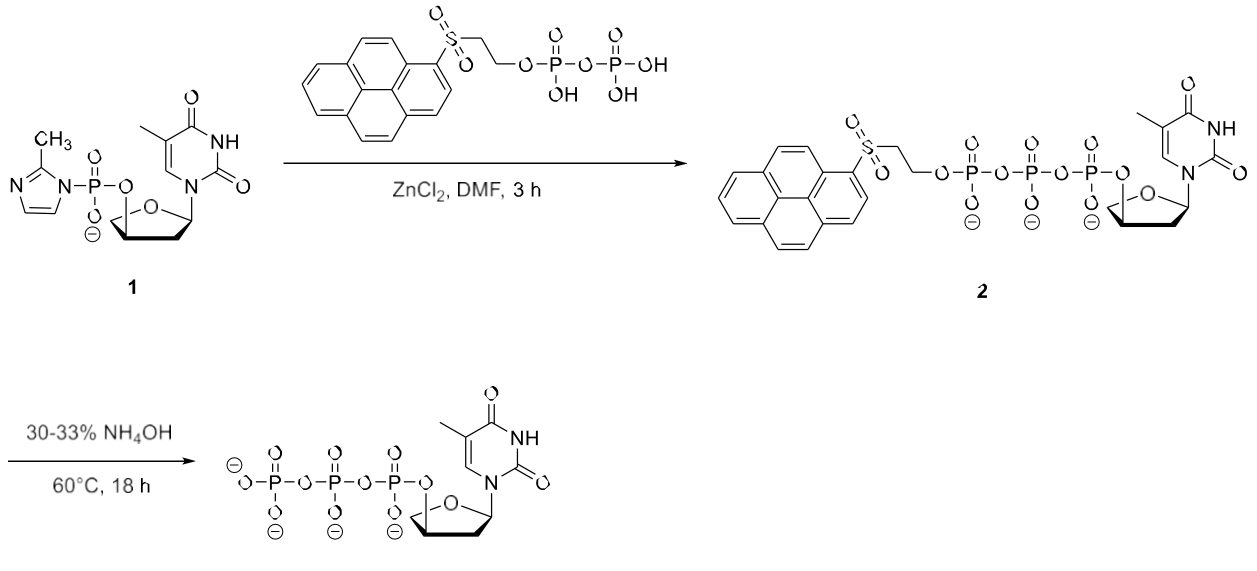

3 (26% two-step yield)

Supplementary Figure 13. **Synthesis of 2′-deoxy-α-L-threofuranosyl thymine triphosphate (dtTTP) by pyrene pyrophosphate method.** Modified synthesis of 2′-deoxy-α-L-threofuranosyl thymine triphosphate (dtTTP) using pyrene pyrophosphate to incorporate the β- and γ-phosphates[2]. The synthesis of compound 1 was reported previously[3].

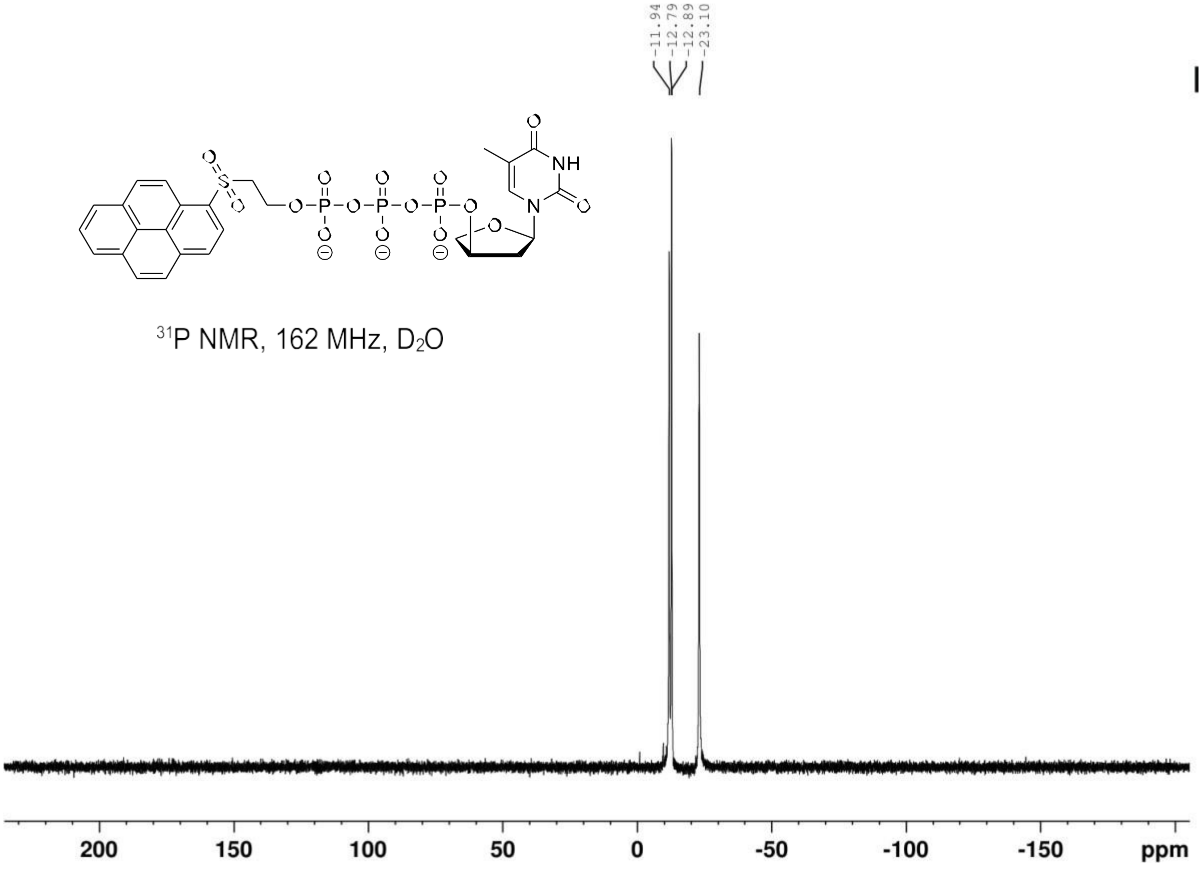

Supplementary Figure 14. NMR spectrum. ^{31}P NMR spectrum of compound 2.

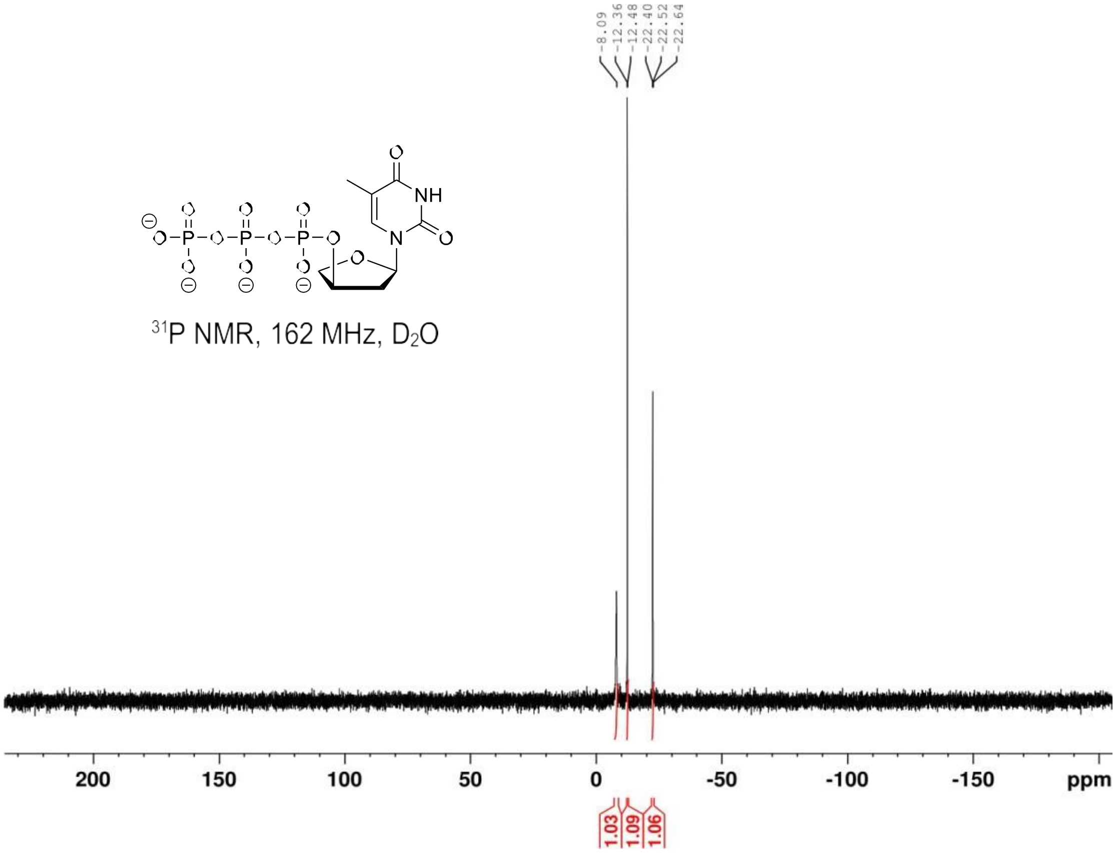

Supplementary Figure 15. NMR spectrum. ^{31}P NMR spectrum of compound 3.

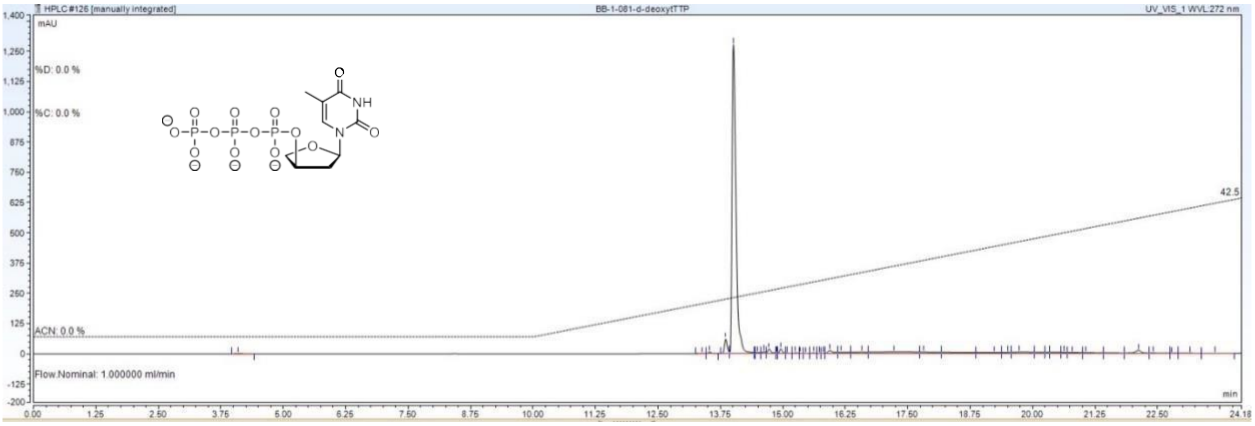

Supplementary Figure 16. HPLC spectrum. HPLC analysis of compound 3.

a

RMSD (Å)	Kod	Kod-RI	5-270	8-64	10-92
Kod		0.540	1.485	1.386	1.227
Kod-RI			1.696	1.623	1.425
5-270				0.356	0.775
8-64					0.774
10-92					

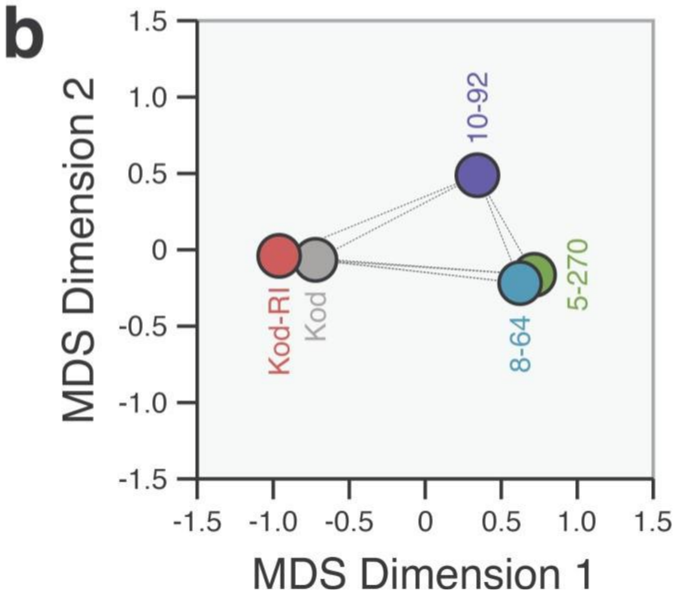

Supplementary Figure 17. Multidimensional analysis of the closed ternary complex of TNAP structures based on RMSD. (a) Pairwise root mean square deviation (RMSD, Å) values for ternary complexes, calculated over Cα atoms following global alignment in PyMOL. Lower RMSD values indicate greater structural similarity. (b) Two-dimensional projection of polymerase relationships derived from the RMSD matrix using classical multidimensional scaling (MDS; double centering + eigendecomposition) in Python (v3.11) using NumPy. Each polymerase is shown as a colored circle. Shorter distances between points on the plot correspond to higher similarity in three-dimensional structure.

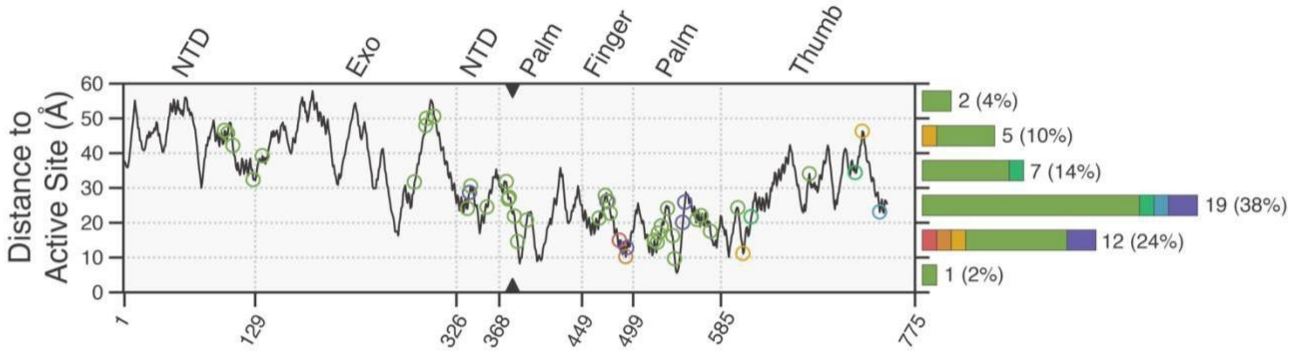

Supplementary Figure 18. Distance of mutations to active site. The distance between the alpha-carbon of each residue in the 10-92 ternary structure to the active site (C2' of the terminal primer TNA residue) is measured in angstroms. Mutations compared to Kod are indicated as open circles and color-matched to Figure 1, with total counts provided in 10 Å shells.

a

```
Binary  Models: Protein (Res 1–775), P 5’-CGCGAACTGC  -3’, T 5’-   AAACGTACGCAGTTCGCG-3’,      2x Mg2+ ions.
Ternary Models: Protein (Res 1–760), P 5’-CGCGAACTGCGT-3’, T 5’-TATGCACGTACGCAGTTCGCG-3’, ATP, 2x Mg2+ ions.
```

b

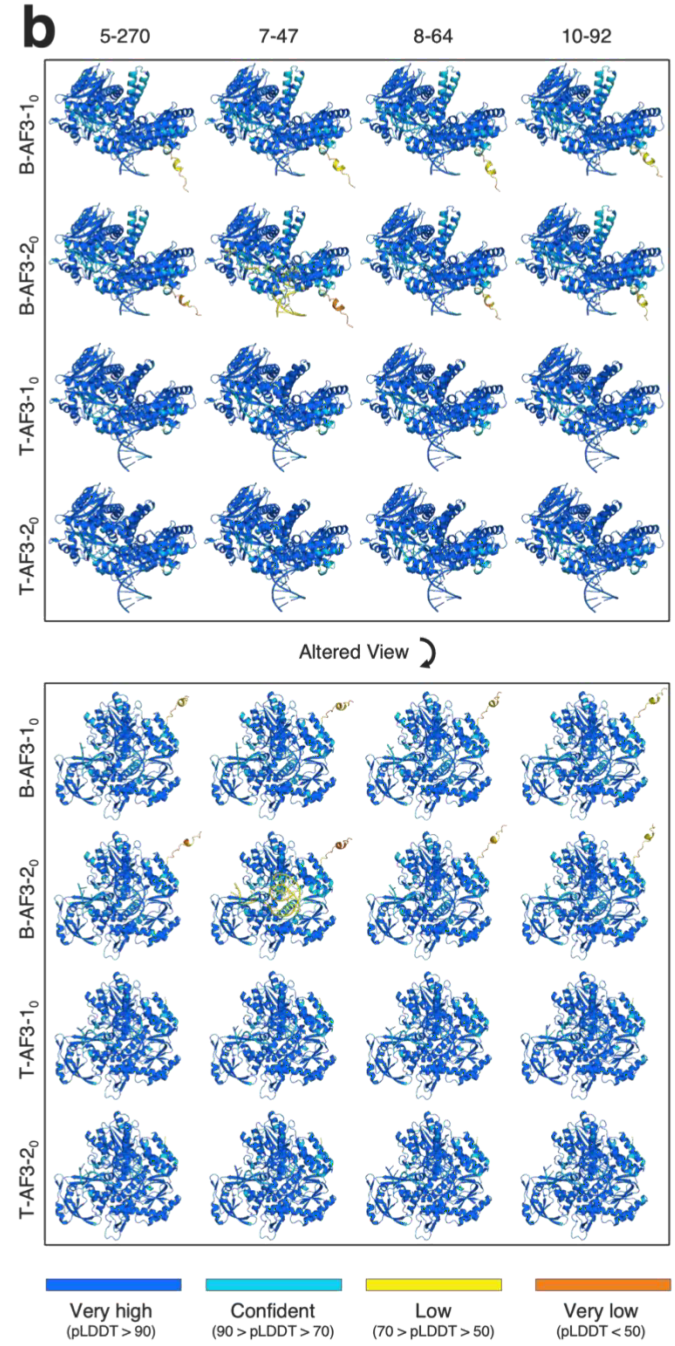

c

TNAP	State	Model Name	ipTM	pTM
5-270	Binary (B)	5B-AF3-1_0	0.95	0.94
		5B-AF3-2_0	0.95	0.94
7-47		7B-AF3-1_0	0.95	0.94
		7B-AF3-2_0	0.73	0.88
8-64		8B-AF3-1_0	0.94	0.94
		8B-AF3-2_0	0.94	0.94
10-92		10B-AF3-1_0	0.95	0.94
		10B-AF3-2_0	0.95	0.94
5-270	Ternary (T)	5T-AF3-1_0	0.94	0.95
		5T-AF3-2_0	0.95	0.95
7-47		7T-AF3-1_0	0.96	0.95
		7T-AF3-2_0	0.95	0.95
8-64		8T-AF3-1_0	0.96	0.95
		8T-AF3-2_0	0.96	0.95
10-92		10T-AF3-1_0	0.97	0.95
		10T-AF3-2_0	0.96	0.95

d

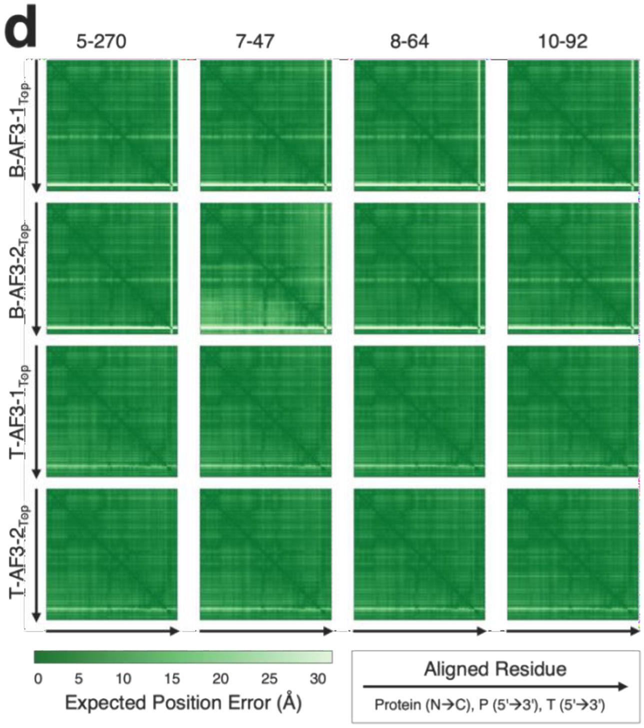

Supplementary Figure 19. AlphaFold3 prediction overview. (**a**) Input specifications for AlphaFold3 binary and ternary complex predictions. Two independent predictions were run for each structure (denoted AF3-1 and AF3-2) with each seed yielding five models (0–4). (**b**) Representative predicted models (model 0) shown in two orientations and colored by per-residue predicted local distance difference test (pLDDT) confidence scores. (**c**) Summary table of predicted interfacial template modeling score (ipTM) and global template modeling score (pTM) for each representative model. (**d**) Predicted alignment error (PAE) matrices shown in Ångströms for the top-ranked model of each run.

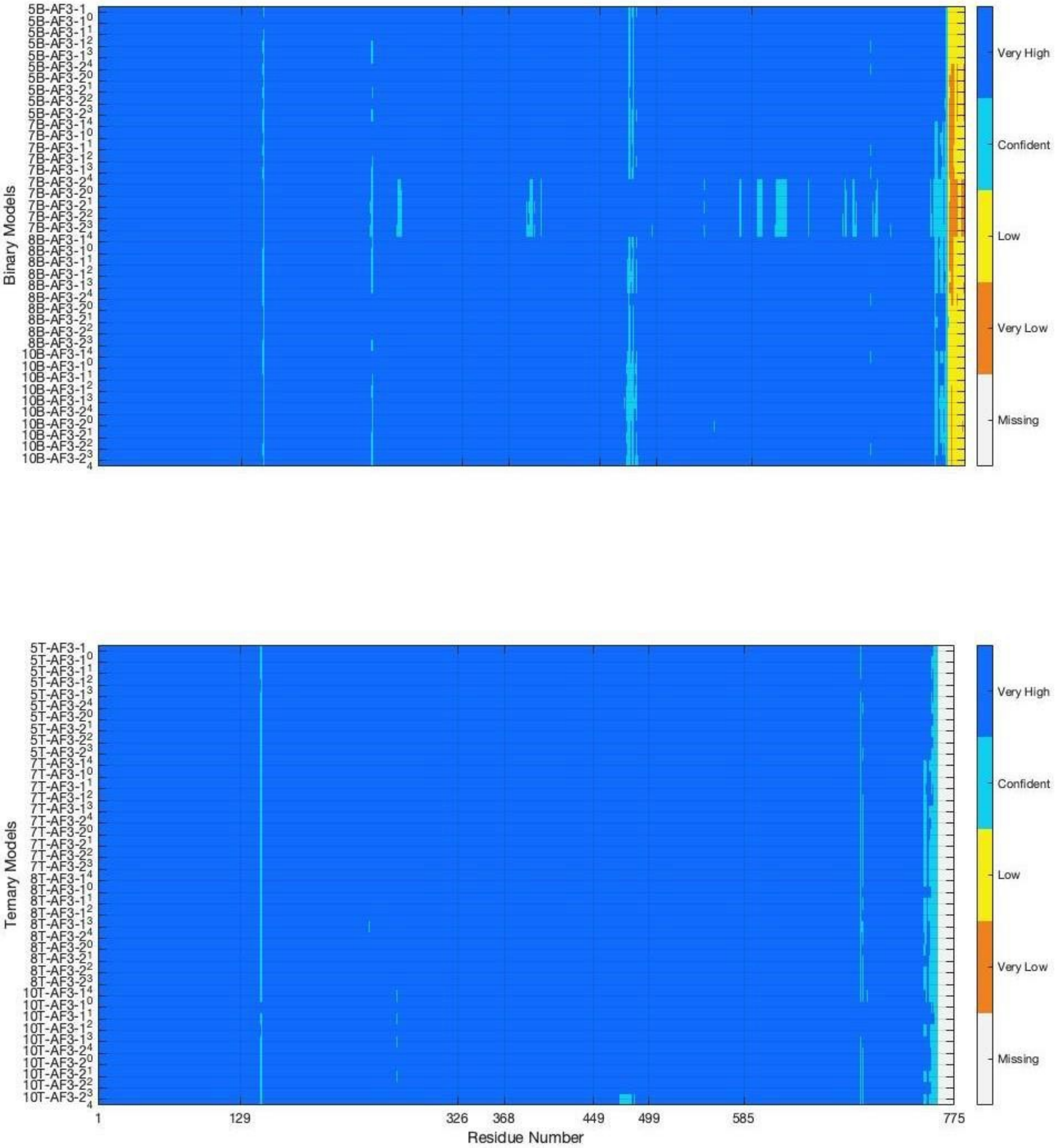

Supplementary Figure 20. AlphaFold3 prediction confidence. AlphaFold3 model confidence (plDDT) heatmaps for binary (top) and ternary (bottom) complexes. Each row represents a single AlphaFold3 model. Two independent AlphaFold3 prediction runs were performed for each structure (denoted AF3-1 and AF3-2), each yielding five models (models 0–4, indicated by subscripts). Residue positions are shown along the x-axis, indicating domain boundaries. Confidence scores were binned as follows: Very high (plDDT > 90, dark blue), Confident (90 > plDDT > 70, light blue), Low (70 > plDDT > 50, yellow), and Very low (plDDT < 50, orange). Residues 761-775 were not predicted for the ternary structures.

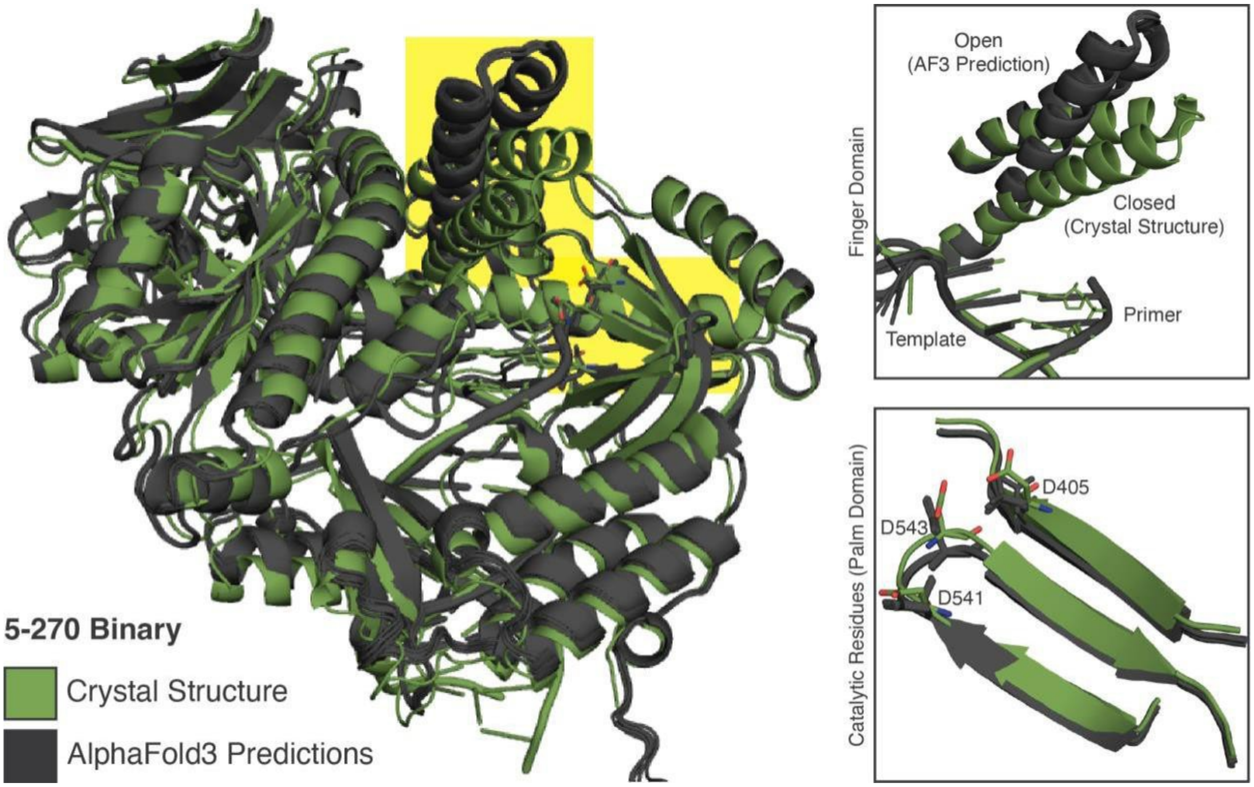

Supplementary Figure 21. AlphaFold3 prediction of the 5-270 binary structure. Overlay of the experimentally determined binary structure (5-270, green) with five AlphaFold3 models (AF3-$1_{0\text{-}4}$, black). Overall structural RMSD = 1.430, 1.461, 1.449, 1.386, and 1.408 Å. AlphaFold3 does not predict the finger domain conformation or the rotamers of the catalytic triad.

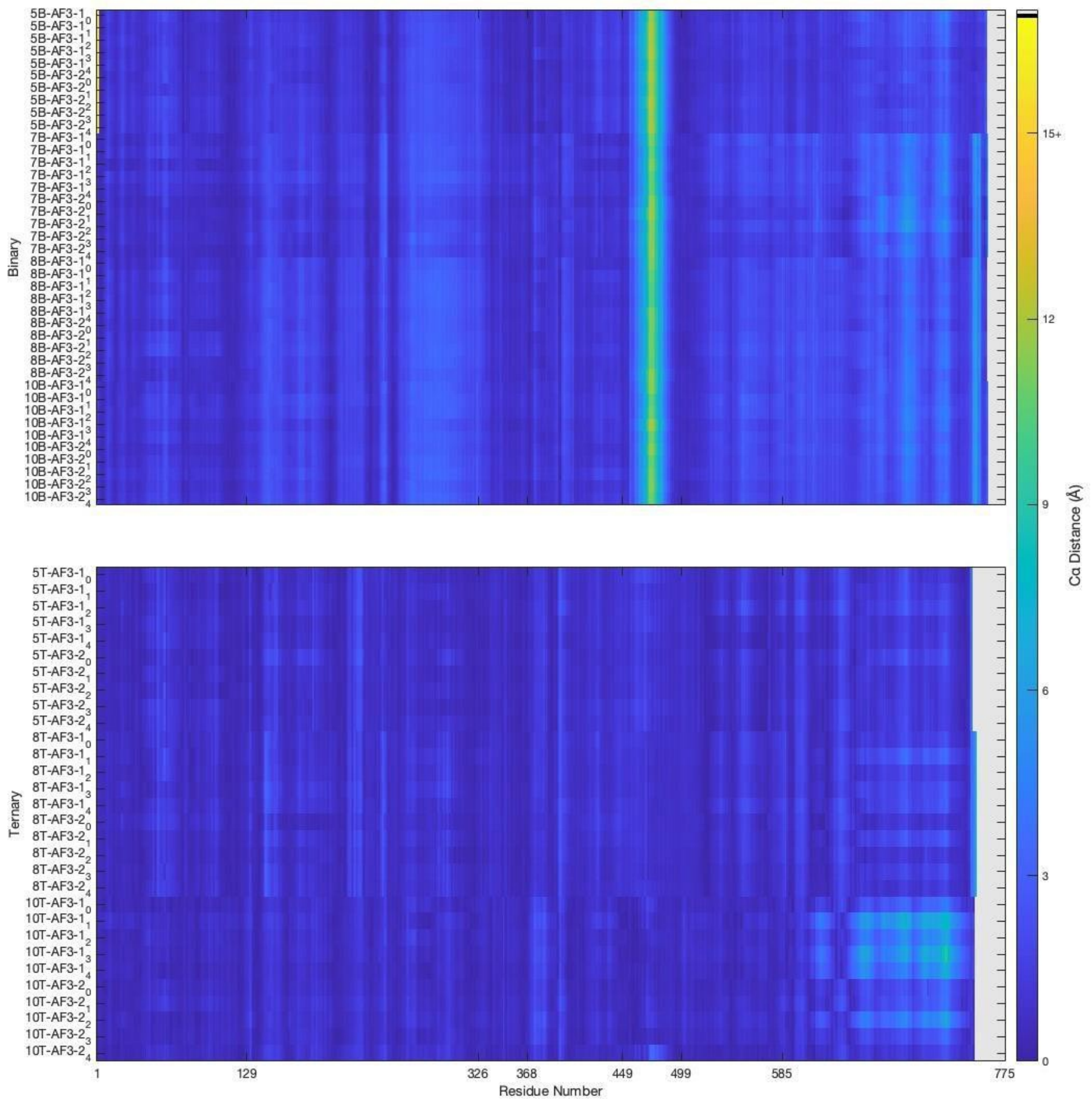

Supplementary Figure 22. Comparison of AlphaFold3 predictions to experimental structures. The top panel shows AlphaFold3 predictions of the binary complexes (5-270, 7-47, 8-64, and 10-92), while the bottom panel shows predictions of ternary complexes (5-270, 8-64, and 10-92). Two AlphaFold3 runs were performed per model (denoted AF3-1 and AF3-2), producing 5 models per run (models 0 – 4, denoted by subscripts). Model predictions (10 per protein) are viewed as a heatmap across the amino acid sequence. Numbering along the backbone denotes domain and subdomain boundaries. Distances are measured between the predicted and crystallographic Cα position for each amino acid residue along the backbone.

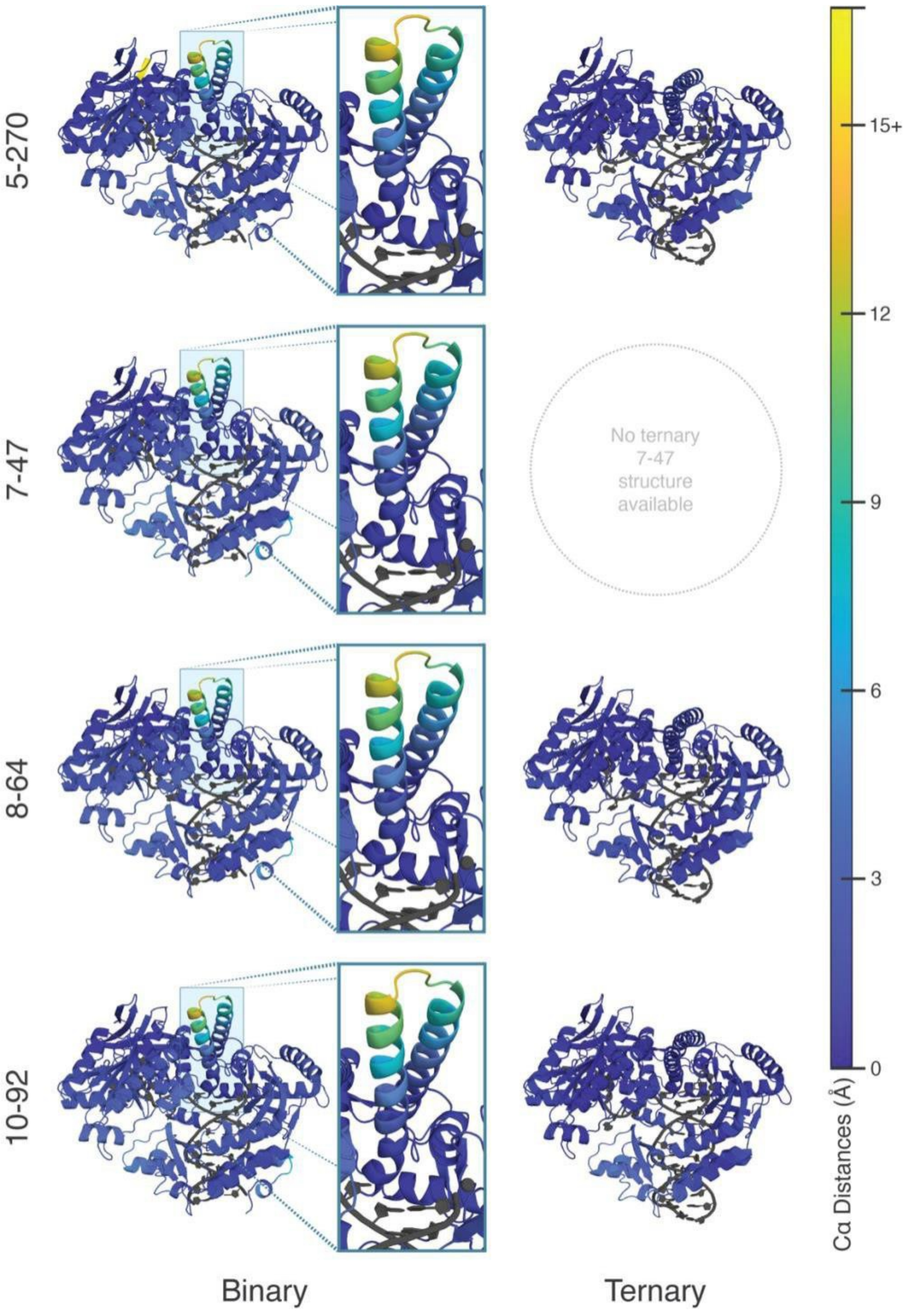

Supplementary Figure 23. Visualizing AlphaFold3 Predictions Against Experimental Structures. Structural validation of AlphaFold3 predictions (AF3-1_0) relative to crystal structures for each crystallized binary and ternary complex (5-270, 7-47, 8-64, and 10-92). AlphaFold3 predictions are shown as cartoons, with residues colored according to the Cα distance (Å) between the predicted and experimentally resolved structures, following the indicated color scale. The zoomed-in view focuses on the binary finger domains. No experimental crystal structure is available for the ternary 7-47 complex for comparison.

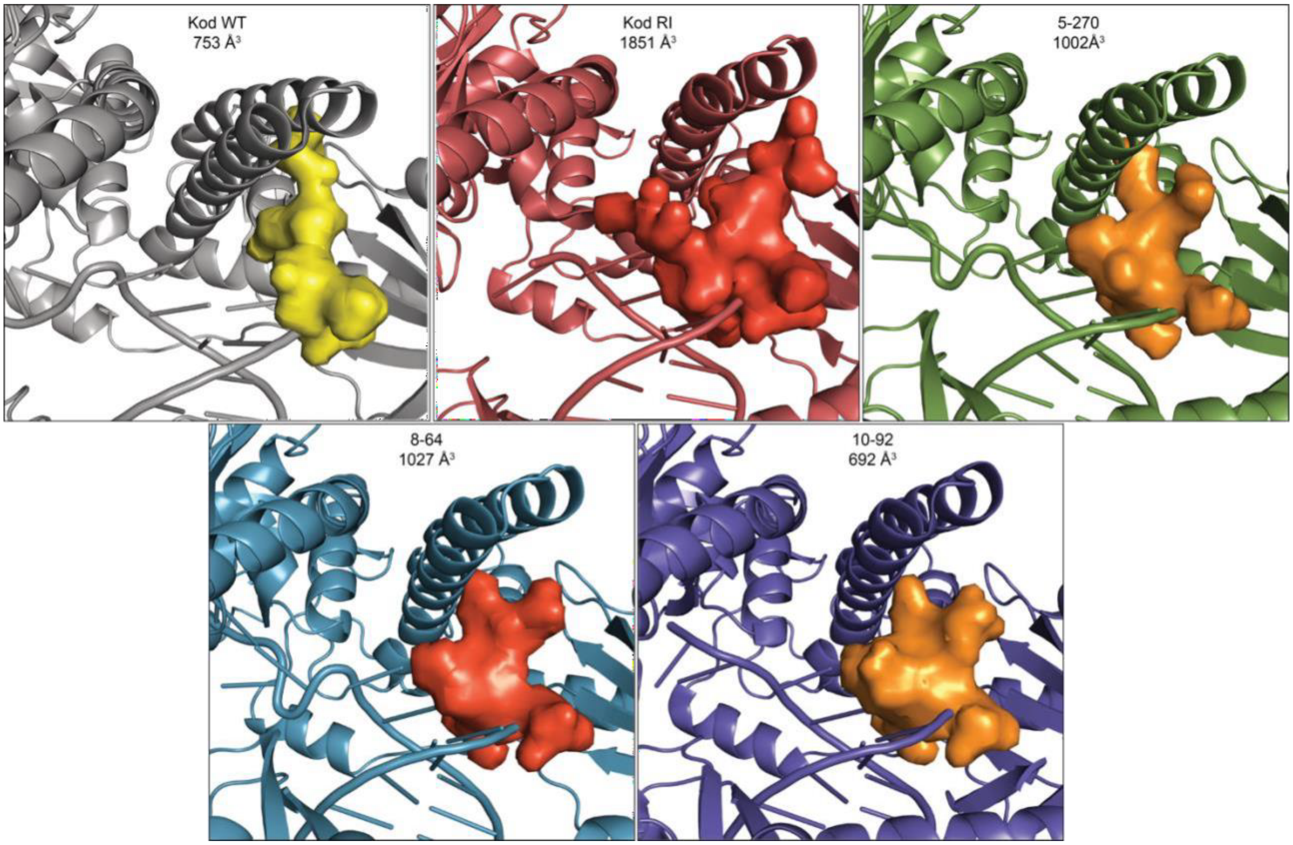

Supplementary Figure 24. Active site pocket volume. Pocket volumes were calculated using PyVol, searching all pockets over a minimum volume threshold (500 Å) without partitioning. Probe min and max radii were set to 1.4 Å and 2.8 Å, respectively. Metals, incoming tNTP triphosphates, and solvents were removed for measurements.

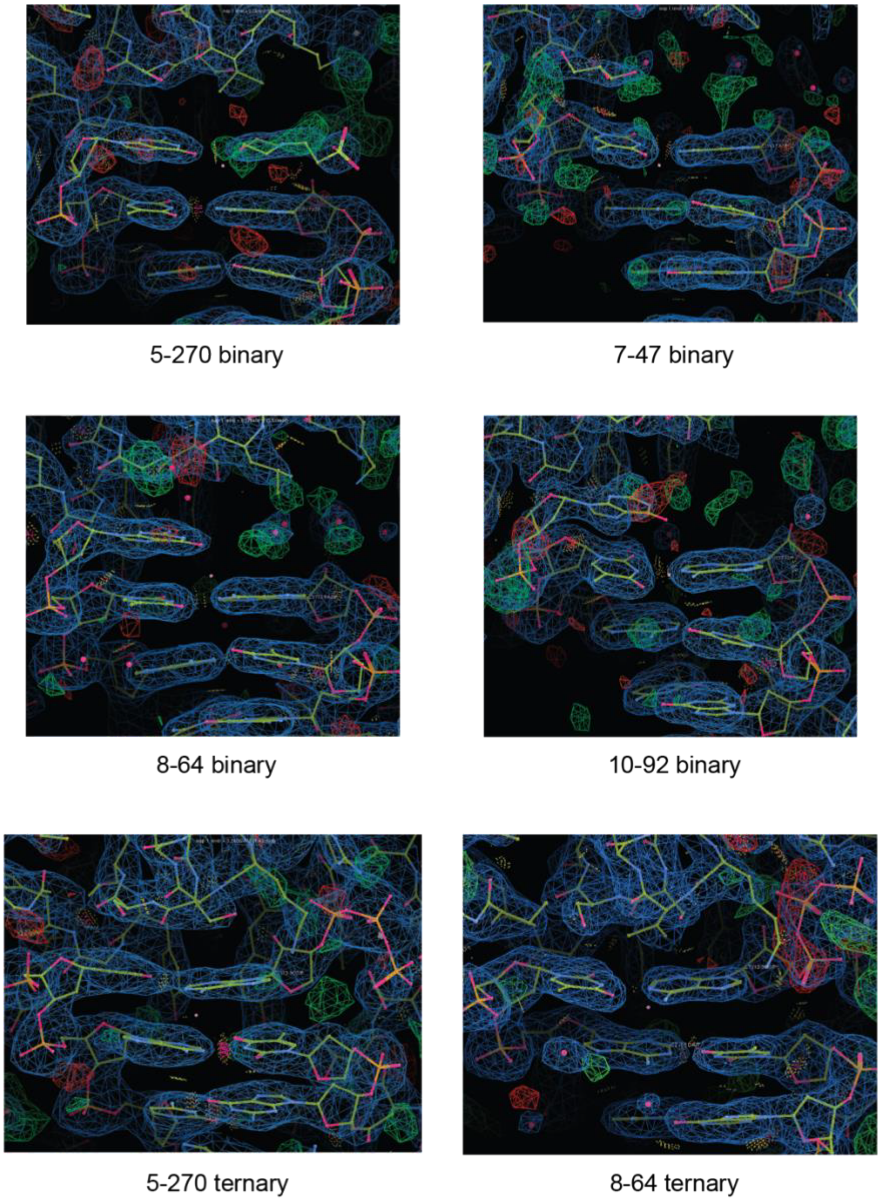

Supplementary Figure 25. 2Fo-Fc electron density maps of the active site. For the newly reported structures, active site 2Fo-Fc electron density maps, contoured at 1.5σ, are shown.

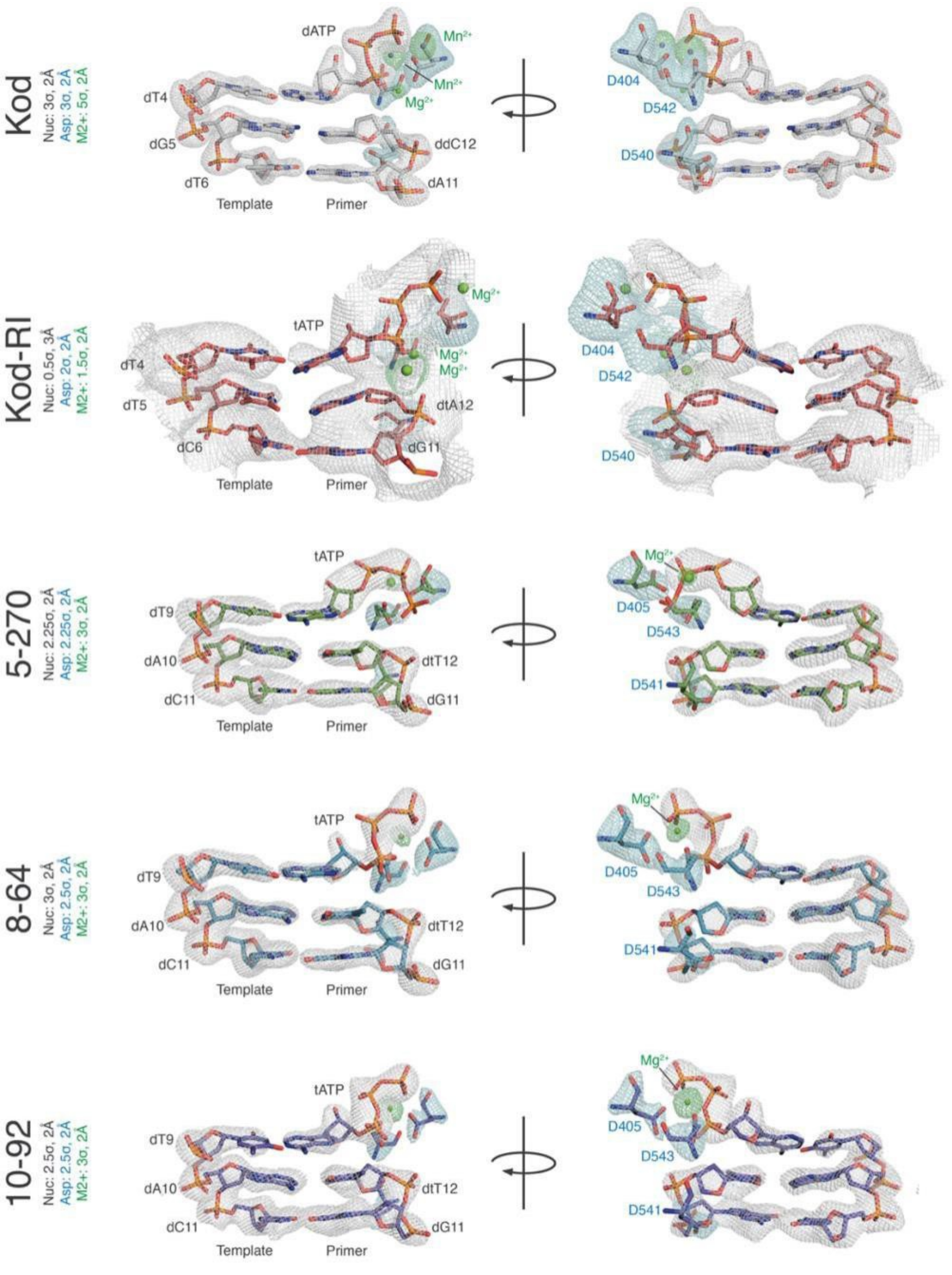

Supplementary Figure 26. Polder maps of key residues in the active site. The incoming triphosphate, neighboring nucleotides, catalytic aspartates (D404/D405, D540/D541, and D542/D543), and divalent metal ions within the active sites of closed ternary complexes are shown. Polder maps (*mFo–DFc*) are displayed (nucleic acids, *Nuc*, gray; catalytic aspartates, *Asp*, aqua; divalent metals, M^{2+}, green), contoured at the indicated σ levels and carved to the specified distances. A 180° rotation about the y-axis provides an alternative view with side chains shown on the right.

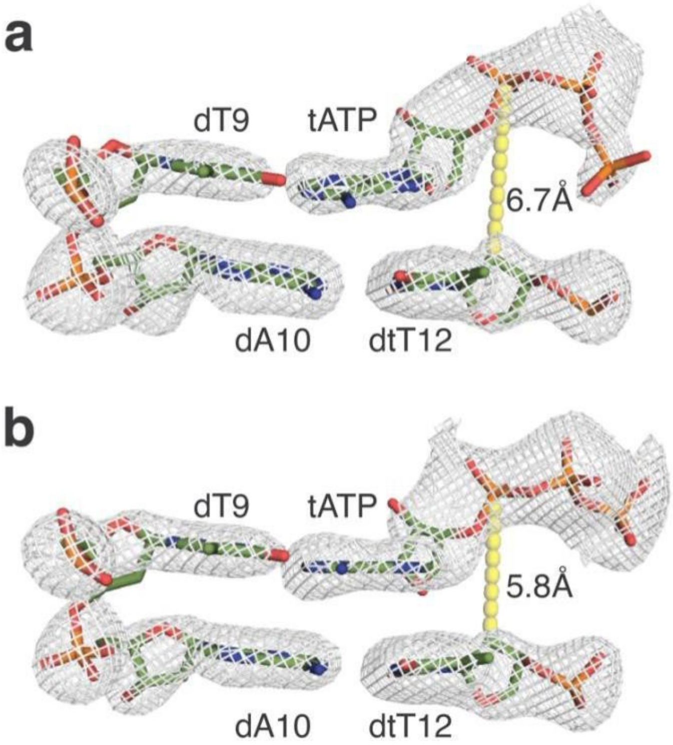

Supplementary Figure 27. **Active site geometry comparing two independently solved structures of 5-270 in a closed ternary complex.** Reaction centers are shown for (**a**) the original crystallographic dataset *G7* and (**b**) an alternative independent dataset *G3*. The distance from the primer to the α-phosphate of the incoming nucleotide is indicated by a dashed yellow line. *mFo–DFc* polder maps support the modeled positions, contoured at 3.0σ and carved to 2.0 Å. The large pocket volume accounts for variability in the orientation of the triphosphate tail.

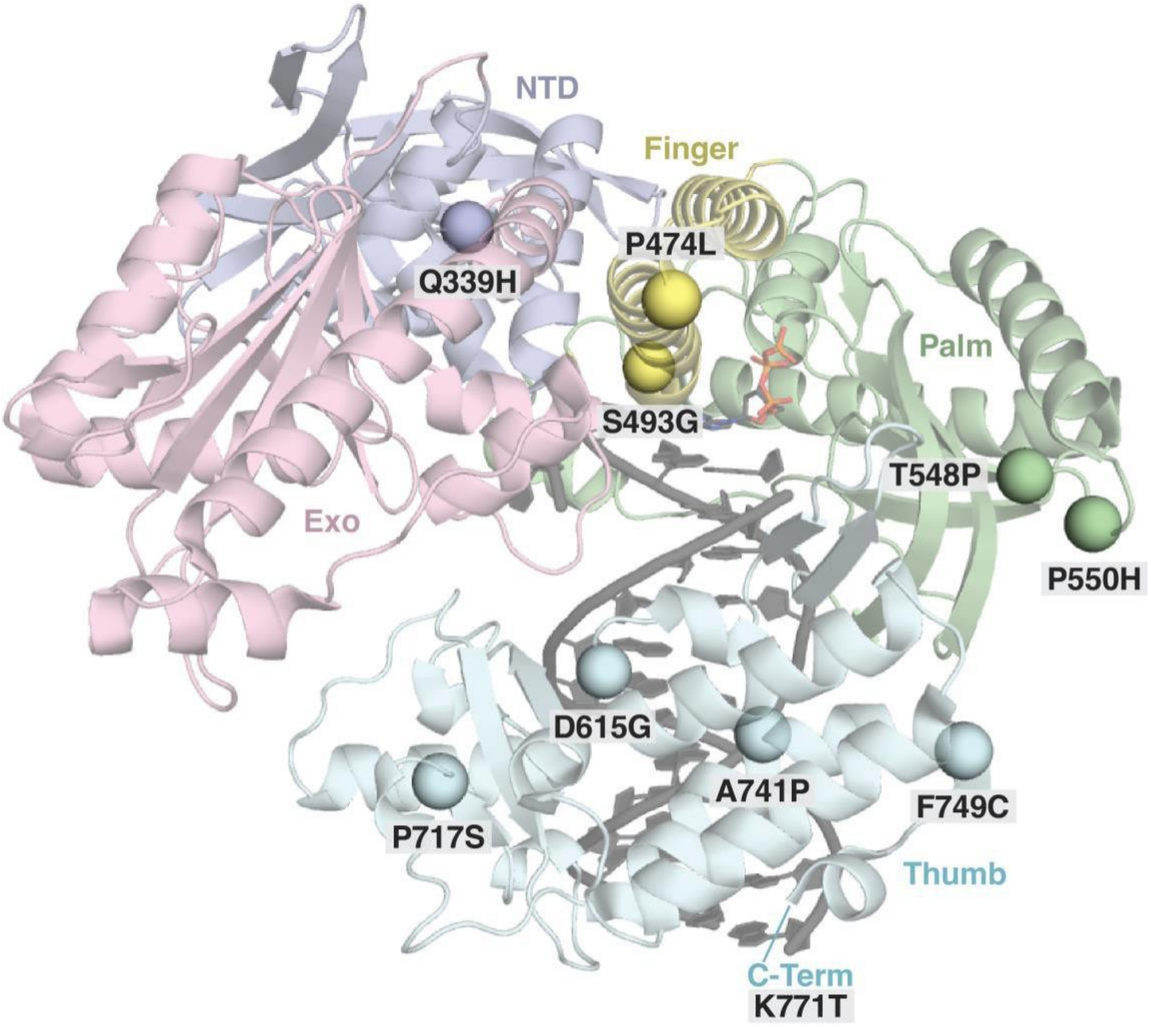

Supplementary Figure 28. Catalysis-enhancing mutations mapped to Kod DNA polymerase. The Kod^{exo-} scaffold (PDB ID 5OMF) is colored by domain. Spheres mark the Cα atoms of the ten residues mutated during error-prone evolution from 5-270 to 10-92. The K771T mutation near the C-terminus was not resolved in the crystal structure and is therefore not depicted. Domains are color-matched to Supplementary Figures 9 and 10.

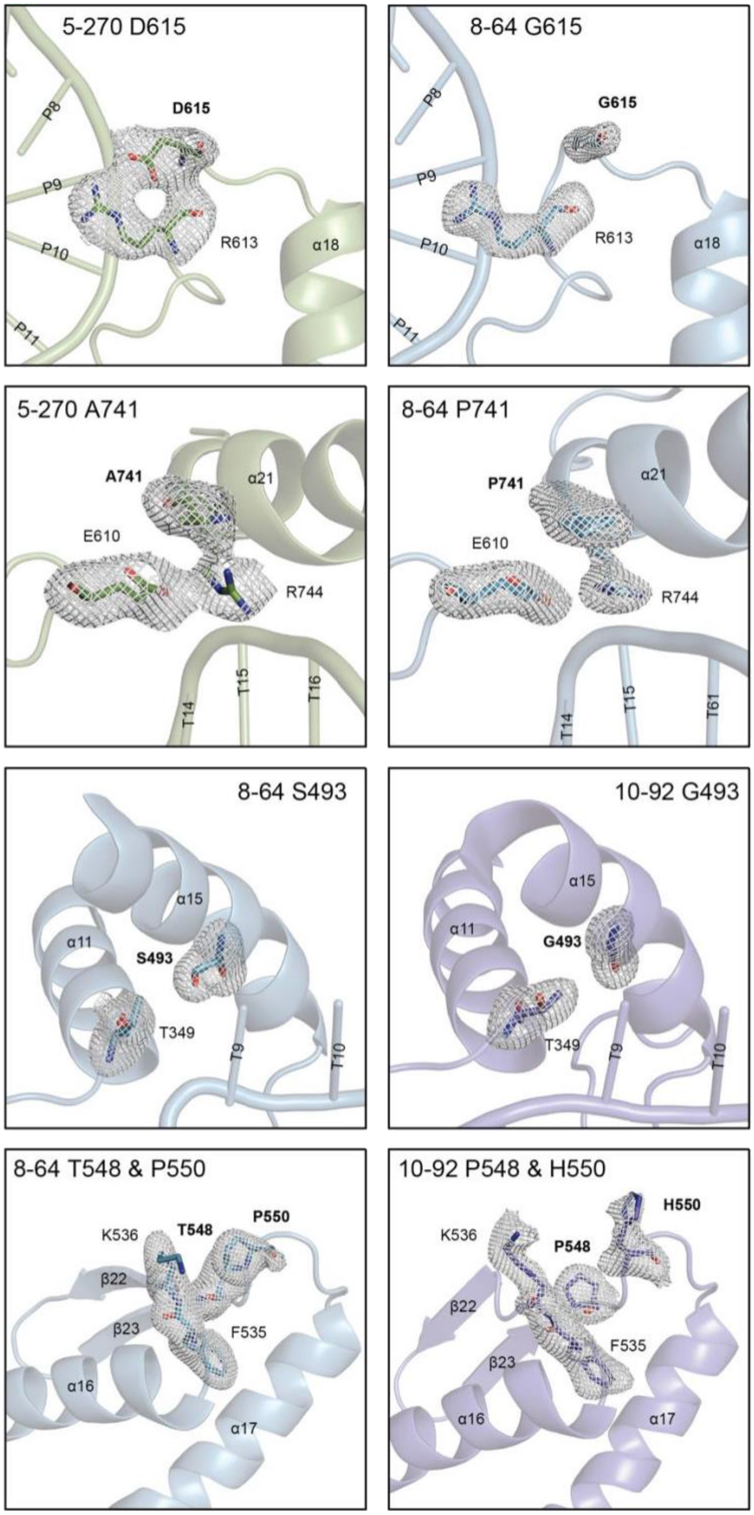

Supplementary Figure 29. Polder maps of mutated residues in 5-270, 8-64, and 10-92. Polder maps (mFo–DFc) are displayed for E610, R613, D615/G615, A741/P741, and R744 in 5-270 and 8-64, and T349, S493/G493 F535, K536, T548/P548, and P550/H550 in 8-64 and 10-92. Residues shown in red indicates key residues that were mutated. S493 and T349 in 8-64 are contoured at 3.5σ and carved to 2.0 Å, while the rest of the maps are contoured at 3.0σ and carved to 2.0 Å.

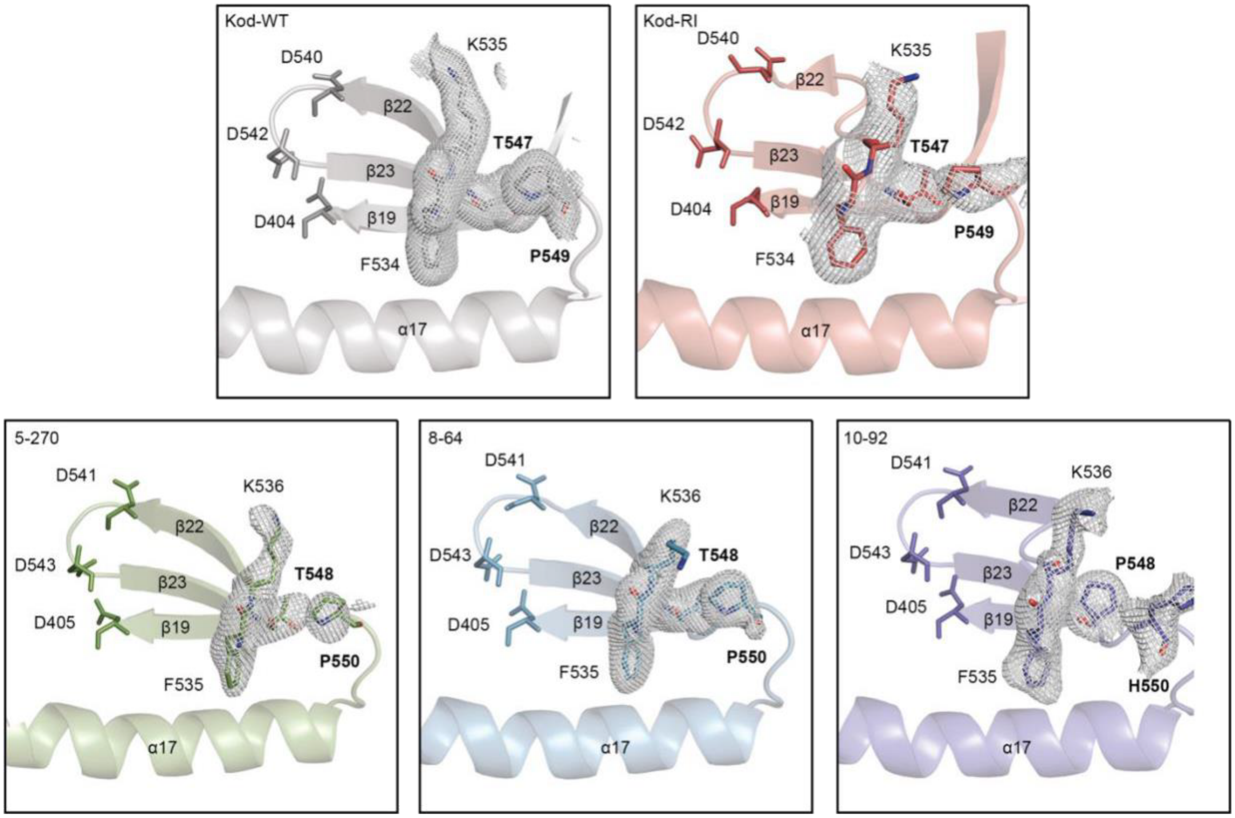

Supplementary Figure 30. Polder maps of mutated residues in DNAP and TNAPs relative to the catalytic aspartate residues. Kod-WT, Kod-RI, 5-270, 8-64, and 10-92 structures are shown in individual panels with polder omit maps (mFo–DFc) for K535/K536, T547/T548/H548, P549/P550/H550. H550 in 10-92 is contoured at 3.5σ and carved to 2.0 Å, 8-64 maps are contoured at 4.0σ and carved to 2.0 Å, and the rest of the maps are contoured at 3.0σ and carved to 2.0 Å.

Supplementary Table 1. DNA oligonucleotides organized by category. Oligonucleotide sequences ordered from IDT written in the 5′→3′ direction. Fluorescent dyes or modifications are noted on the specified termini. Highlighted in cyan and green are NdeI and NotI recognition sequences and in red are mismatched positions for sequences used in fidelity experiments.

	Oligo name	Oligo sequence
**Kinetics**	PBS8 primer	GTCCCCTTGGGGATACCACC
	EM619 template	CCCACACCCTCCTATCGCTAAACACACACTTAATAAAGTTGGTGGTATCCCCAAGGGGAC
**Fidelity**	Acrydite PBS8 primer	/5Acryd/GTCCCCTTGGGGATACCACC
	4NT.9G Fidelity template	GGATCGTCAGTGCAAAGAGATTAAGACTCGCCATGTTACGATCTGCCAAGTACAGCCTTGA ATCGTCACTGGTGGTATCCCCTTGGGGAC
	PBS7.PBS9 overhang primer	CTTTTAAGAACCGGACGAACGGATCGTCAGTGCATTGAGA
	PBS8 primer	GTCCCCTTGGGGATACCACC
	PBS9 primer	CTTTTAAGAACCGGACGAAC
**Thermal challenge**	PBS8_short primer	/5IRD680/GTCCCCTTGG
	EM619 template	CCCACACCCTCCTATCGCTAAACACACACTTAATAAAGTTGGTGGTATCCCCAAGGGGAC
**Cloning**	TNAP-Fwd	ATC*CATATG*ATCCTCGACACTGACTAC
	TNAP-Rvs	ACGCATG*CGGCCGC*TCAAGTTCCTTTCGGCGTCAG
	TNAP-Rvs_760	ACGCATG*CGGCCGC*TCACTTCTGGTAGCGCAGGTC
	8-64-Rvs_760	ACGCATG*CGGCCGC*TCAAGTTCCTTTCGGCGTCAG
**Crystallography**	P	CGCGAACTGCG
	T1	/5Cy5/AAACGTACGCAGTTCGCG
	T2	TATGCACGTACGCAGTTCGCG

Supplementary Table 2. Data collection and refinement statistics for binary structures

	5-270	7-47	8-64	10-92
**Data Collection**				
Space group	$P2_12_12_1$	$P2_12_12_1$	$P2_12_12_1$	$P2_12_12_1$
Cell Dimensions				
a, *b*, *c* (Å)	86.8, 110.5, 124.3	87.2, 110.6, 124.3	86.4, 108.0, 123.6	86.4, 108.3, 123.8
α, β, γ (º)	90.0, 90.0, 90.0	90.0, 90.0, 90.0	90.0, 90.0, 90.0	90.0, 90.0, 90.0
Resolution (Å)	45.95-2.56 (2.652-2.56)	50.51-2.18 (2.258-2.18)	49.5-2.5 (2.589-2.5)	33.3-2.172 (2.25-2.172)
R_{merge}	0.093 (0.863)	0.070 (0.265)	0.1368 (0.8707)	0.1051 (2.163)
CC1/2	0.998 (0.773)	0.99 (0.772)	0.992 (0.667)	0.998 (0.365)
$I / \sigma I$	15.08 (2.09)	10.94 (4.46)	8.39 (2.97)	10.73 (0.96)
Completeness (%)	99.42 (99.87)	98.23 (99.39)	99.91 (99.55)	99.79 (99.13)
Redundancy	6.5 (6.5)	3.4 (3.5)	5.2 (4.3)	6.8 (7.2)
**Refinement**				
Resolution (Å)	2.56	2.18	2.5	2.17
No. reflections	38976 (3841)	62205 (6183)	40682 (3980)	61779 (6048)
R_{work}/R_{free}	0.2181/0.2682 (0.2874/0.3524)	0.1887/0.2259 (0.2174/0.2723)	0.2295/0.2556 (0.3503/0.3649)	0.2167/0.2474 (0.3120/0.578)
No. atoms	6897	7358	6982	6966
Protein/DNA	6874	6873	6875	6878
Ligand	18	45	3	4
Solvent	5	440	104	84
B-factors	60.95	27.13	54.18	61
Protein/DNA	59.33	26.40	52.77	59.21
Ligand	78.70	36.86	47.08	63
Solvent	61.14	27.77	49.26	51.96
R.m.s deviations				
Bond lengths (Å)	0.002	0.003	0.002	0.002
Bond angles (º)	0.46	1.01	0.52	0.47

*Values in parentheses are for the highest-resolution shell.

Supplementary Table 3. Data collection and refinement statistics for ternary structures

	5-270	8-64
**Data Collection**		
Space group	$P2_12_12_1$	$P2_12_12_1$
Cell Dimensions		
a, *b*, *c* (Å)	77.4, 101.7, 110.8	77.7, 101.5, 110.7
α, β, γ (º)	90.0, 90.0, 90.0	90.0, 90.0, 90.0
Resolution (Å)	48.67-3.03 (3.14-3.03)	48.59-2.38 (2.47-2.38)
R_{merge}	0.1628 (1.017)	0.0734 (0.7658)
CC1/2	0.995 (0.769)	0.999 (0.842)
$I / \sigma I$	12.57 (2.31)	19.45 (2.27)
Completeness (%)	99.61 (99.71)	99.87 (99.86)
Redundancy	6.6 (7.0)	6.6 (6.8)
**Refinement**		
Resolution (Å)	3.03	2.38
No. reflections	17539 (1729)	35755 (3519)
R_{work}/R_{free}	0.2240/0.2774 (0.2979/0.3730)	0.2159/0.2655 (0.2640/0.3218)
No. atoms	6783	6875
Protein/DNA	6743	6775
Ligands	41	32
Solvent	5	68
B-factors	73.16	59.48
Protein/DNA	71.59	57.79
Ligands	70.26	61.07
Solvent	47.47	45.98
R.m.s deviations		
Bond lengths (Å)	0.002	0.003
Bond angles (º)	0.39	0.53

*Values in parentheses are for the highest-resolution shell.

Supplementary Table 4. Base pair parameters. Base pair parameters of duplexes in ternary structures computed using Web x3DNA.

**Kod-WT**	Shear	Stretch	Stagger	Buckle	Prop-Tw	Opening	Shift	Slide	Rise	Tilt	Roll	Twist
T-a	-0.01	-0.236	0.064	-5.456	0.746	2.845	0	0	0	0	0	0
G-C	-0.271	-0.217	0.012	-8.368	-14.078	1.971	-0.605	-1.704	3.301	3.044	5.375	29.301
T-A	-0.041	-0.072	-0.218	1.349	0.959	-2.94	-0.741	-1.059	3.181	3.988	3.466	30.229
G-C	-0.374	-0.207	-0.253	-4.65	-1.784	2.365	0.375	-0.76	3.618	-3.476	0.867	37.528
G-C	-0.161	-0.058	-0.284	-12.984	-5.434	2.706	0.19	-1.105	3.634	1.913	3.746	30.239
C-G	-0.003	-0.181	0.283	-4.669	-7.263	-2.415	-1.05	-0.114	3.237	-5.455	1.91	32.406
C-G	0.444	-0.198	0.172	6.097	-8.909	2.69	0.322	0	3.125	1.314	9.18	29.577
G-C	-0.12	-0.238	0.582	6.321	-20.192	0.946	-0.973	0.556	3.334	-7.284	4.185	33.328
T-A	-0.22	-0.229	0.685	-7.786	-15.669	0.753	-0.196	-0.659	3.661	-0.31	-1.082	34.762
G-C	0.337	-0.07	0.356	8.874	-7.834	0.832	0.782	0.587	2.988	3.285	-0.55	36.967
G-C	-0.459	-0.047	0.702	-2.255	-18.78	4.552	0.18	-0.546	3.416	-3.949	3.155	31.512
T-A	0.108	-0.157	-0.043	-3.203	-16.523	1.076	-0.764	-0.964	3.225	6.71	0.883	35.98
C-G	0.125	-0.241	-0.341	6.525	-2.221	-2.727	0.796	-0.399	3.142	3.83	1.518	35.085
**Kod-RI**	Shear	Stretch	Stagger	Buckle	Prop-Tw	Opening	Shift	Slide	Rise	Tilt	Roll	Twist
T-a	0.025	-0.713	-1.831	-20.004	-20.512	-15.264	0	0	0	0	0	0
T-a	-1.15	-0.098	-0.315	-32.927	-8.414	-6.13	-0.248	-0.412	4.146	-9.214	4.829	22.767
C-G	0.548	-0.465	0.194	-12.395	8.368	4.839	0.292	0.169	3.187	-7.214	10.287	33.488
G-C	0.219	-0.089	-0.245	-8.262	-5.838	6.681	-0.417	-0.654	3.479	4.25	-6.934	35.888
C-G	-0.074	0.94	0.649	-11.417	-8.149	25.954	1.297	-1.113	3.282	-3.717	3.475	29.268
A-T	1.05	-0.208	-0.295	12.854	-2.334	-2.286	-1.941	0.944	2.978	6.559	0.132	33.195
G-C	-0.095	-0.298	1.19	8.051	4.134	10.399	1.033	-1.502	3.404	-14.843	8.355	24.019
T-A	-0.54	-0.412	0.415	6.18	-20.716	-0.975	-1.07	-0.709	3.533	9.595	-2.63	34.032
T-A	0.682	-0.426	0.416	1.218	-15.606	-1.074	0.382	-0.319	3.342	1.396	3.136	38.543
C-G	0.346	-0.028	-0.264	20.634	-9.77	6.291	1.103	0.339	2.729	12.053	5.085	32.118
C-G	-0.998	1.108	-1.466	18.676	-42.087	-49.397	-1.416	0.893	6.23	7.057	0.978	57.702
**7-47**	Shear	Stretch	Stagger	Buckle	Prop-Tw	Opening	Shift	Slide	Rise	Tilt	Roll	Twist
T-a	0.581	-0.161	-0.632	-14.01	3.651	3.729	0	0	0	0	0	0
A-t	-0.33	-0.316	0.264	-12.001	-15.878	2.545	-1.226	-1.453	3.454	-4.866	7.898	20.418
C-G	-0.281	-0.279	0.006	-5.25	0.447	-1.935	-0.664	-0.735	3.2	4.482	5.76	30.569
G-C	-0.338	-0.137	-0.001	-6.306	-16.368	5.687	0.297	-0.228	3.497	-1.32	10.256	35.117
C-G	0.501	-0.301	0.122	-10.195	2.838	3.516	0.03	-0.151	3.435	-1.238	0.731	34.881
A-T	0.344	-0.195	0.611	6.511	-2.481	-8.074	-1.278	0.429	3.038	-5.326	-3.241	33.223
G-C	0.101	-0.098	0.339	10.127	-4.82	9.088	1.287	-0.677	3.402	1.064	8.808	27.68
T-A	-0.367	-0.281	0.277	6.647	-16.741	-0.137	-1.096	-0.549	3.46	0.829	1.387	31.864
T-A	-0.307	-0.271	0.207	5.235	-16.374	-4.05	-0.099	-0.262	3.305	1.078	2.785	37.91
C-G	0.267	-0.345	-0.027	-0.78	-12.303	-2.07	0.486	-0.059	3.408	4.085	6.795	36.894
G-C	0.007	-0.32	-0.246	-2.119	-7.847	0.003	0.344	0.451	3.396	3.327	7.686	32.664
C+G	-0.108	-5.304	0.071	4.607	-0.38	81.91	-2.188	3.315	-0.894	174.391	-12.494	-138.25
G-C	-0.971	-1.128	-2.394	-34.185	-21.806	12.434	2.203	3.984	-0.606	-122.9	93.499	-113.624
**8-64**	Shear	Stretch	Stagger	Buckle	Prop-Tw	Opening	Shift	Slide	Rise	Tilt	Roll	Twist
T-a	0.552	-0.225	-0.205	-13.85	1.559	2.082	0	0	0	0	0	0
A-t	-0.052	-0.301	0.03	-10.223	-19.876	8.119	-0.812	-1.39	3.417	1.555	8.403	23.644
C-G	-0.005	-0.165	-0.383	-4.068	-2.314	0.284	-0.666	-0.339	3.261	6.477	4.187	30.737
G-C	0.025	0.072	0.117	-6.053	-16.221	6.737	-0.021	0.13	3.527	-4.558	17.572	35.08
C-G	0.339	-0.237	-0.339	-4.062	1.166	4.925	0.408	-0.184	3.315	2.719	-1.819	32.071
A-T	-0.016	-0.097	0.207	5.26	-10.203	-2.972	-1.429	0.469	3.24	-5.885	0.557	32.074
G-C	-0.109	-0.219	-0.216	-2.347	-11.83	5.865	0.705	-0.399	3.728	0.517	11.798	29.169
T-A	-0.387	-0.332	-0.394	7.021	-17.831	2.441	-1.074	-0.471	3.044	-0.022	4.633	29.771
T-A	-0.437	-0.317	-0.071	5.587	-21.638	-0.932	-0.207	0.012	3.3	-1.832	5.356	37.027
C-G	0.658	-0.388	-0.137	1.485	-11.061	1.957	0.645	0.167	3.38	1.752	4.017	38.519
G-C	-0.858	-0.418	-0.444	-15.041	-16.907	5.539	0.239	0.684	3.668	3.817	12.95	31.843
C-G	0.6	-0.244	-0.226	6.755	-3.96	-1.913	-0.275	0.364	2.753	-4.524	5.717	31.987
G-C	-1.193	-0.897	-2.257	-35.235	-27.081	9.202	0.705	1.646	4.511	15.653	15.696	33.765
**10-92**	Shear	Stretch	Stagger	Buckle	Prop-Tw	Opening	Shift	Slide	Rise	Tilt	Roll	Twist
T-a	0.437	-0.352	-0.884	-5.879	-1.4	1.627	0	0	0	0	0	0
A-t	-0.107	0.062	0.258	-18.035	-18.371	5.27	-0.329	-1.356	3.743	-4.419	15.703	24.875
C-G	0.035	-0.152	0.067	-8.551	-0.961	-1.952	-0.594	-0.46	3.188	5.43	5.21	31.003
G-C	0.065	-0.003	-0.097	-18.373	-10.071	7.339	0.26	-0.094	3.684	0.568	10.385	36.021
C-G	0.531	0.081	-0.366	-3.079	-6.454	9.812	0.646	0.23	2.957	5.087	4.259	31.317
A-T	0.112	-0.073	0.493	0.763	-5.571	-6.963	-1.663	0.642	3.345	-11.641	3.443	32.906
G-C	-0.175	0.293	0.501	2.177	-8.584	12.314	0.807	-0.827	3.286	-2.303	5.462	27.833
T-A	-0.104	-0.199	0.404	1.139	-11.845	-2.138	-1.442	-0.842	3.446	-0.208	3.022	31.388
T-A	-0.184	-0.164	-0.402	9.206	-26.265	-2.821	-0.231	0.003	2.909	5.907	-0.747	36.739
C-G	0.181	-0.041	0.496	-3.26	-18.176	3.541	0.626	-0.19	3.547	-3.181	10.773	34.391
G-C	0.55	0.157	0.168	-9.408	-28.969	-4.303	-0.405	0.423	3.499	5.018	11.005	38.193
C-G	0.425	-0.062	0.057	-16.986	2.121	6.174	1.471	0.568	3.542	1.889	3.763	34.259
G-C	0.304	-0.117	0.091	4.935	-10.879	-1.552	-0.678	1.743	3.147	-2.838	-0.161	35.274

Supplementary Table 5. Integrated mutational analysis. For each mutation, the polymerase domain, generation of appearance during TNAP evolution, BLOSUM62 substitution score, predicted stability change (FoldX ΔΔG, kcal mol^{-1}), Cα distance to the active site (Å), and absolute and relative solvent-accessible surface area (ABS SASA, $Å^2$; REL SASA, % of maximum) are reported for the ternary structures of Kod^{exo-}, 5-270, and 10-92. Functional impact is summarized as residual activity (% of 10-92) upon reversion to 5-270, extracted from prior data[1]. N/A = non-applicable.

Mutation	Domain	TNAP Generation	BLOSUM62	FoldX ΔΔG (kcal mol−1)	Cα Distance to Active Site (Å)			ABS SASA (Å^2)			REL SASA (% of max)			% Activity if reverted	
					Kod	5−270	10−92	Kod	5−270	10−92	Kod	5−270	10−92	45−sec Reaction	60−sec Reaction
K99R	NTD	5−270	2	0.061	47.5	46.8	46.6	103.1	130.5	144.0	50.3	54.8	60.5		
E102A	NTD	5−270	−1	0.016	46.5	45.2	45.8	132.3	86.3	76.4	75.9	79.3	70.2		
I107V	NTD	5−270	3	0.650	43.4	42.6	42.3	97.5	73.3	68.1	55.5	48.2	44.8		
V127I	NTD	5−270	3	−0.640	32.6	33.3	32.4	31.9	55.1	36.9	21.0	31.4	21.0		
K136T	Exo	5−270	−1	1.173	39.8	39.8	39.3	95.6	67.0	51.9	46.6	47.7	36.9		
D141A	Exo	Kodexo−	−2	N/A	38.7	39.1	38.6	13.4	12.0	11.8	12.3	11.1	10.8		
E143A	Exo	Kodexo−	−1	N/A	42.6	42.8	42.4	5.2	8.8	9.1	4.8	8.0	8.4		
Q285K	Exo	5−270	1	−0.323	31.8	31.2	31.7	103.1	97.2	124.9	57.7	47.4	60.9		
T296A	Exo	5−270	0	1.125	47.7	47.9	48.0	9.3	34.5	11.9	6.6	31.7	11.0		
T297Q	Exo	5−270	−1	−0.260	49.8	49.9	50.1	77.1	85.2	130.0	54.8	47.6	72.7		
N304G	Exo	5−270	0	−0.815	51.2	50.7	50.7	97.5	38.6	26.5	67.3	47.6	32.7		
I337V	NTD	5−270	3	0.916	24.6	25.4	24.0	0.2	3.2	1.8	0.1	2.1	1.2		
Q339H	NTD	33878	0	1.633	29.2	29.9	28.7	0.5	2.7	2.7	0.3	1.5	1.5	90.20	93.62
S340P	NTD	5−270	−1	−1.031	30.7	31.8	30.5	1.3	2.6	0.2	1.1	1.9	0.1		
F356Y	NTD	5−270	3	0.779	24.5	25.1	24.5	8.0	42.1	40.4	4.0	19.6	18.9		
K375R	Palm	5−270	2	−0.275	33.4	31.6	31.8	160.1	150.4	144.1	78.1	63.1	60.5		
L377Y	Palm	5−270	−1	1.387	28.1	26.7	26.8	25.0	45.7	51.1	13.9	21.3	23.9		
A378E	Palm	5−270	−1	−0.214	29.1	27.1	27.3	74.4	56.7	103.7	68.4	32.5	59.6		
+381L	Palm	5−270	N/A	N/A	N/A	22.1	22.6	N/A	102.3	94.7	N/A	57.0	52.7		
Q383E	Palm	5−270	2	0.076	22.5	21.7	21.9	100.3	41.8	43.4	56.1	24.0	24.9		
E386A	Palm	5−270	−1	0.822	15.0	14.2	14.5	96.8	54.8	52.5	55.5	50.4	48.3		
R395K	Palm	5−270	2	−0.061	18.9	20.8	20.6	132.6	116.1	110.0	55.7	56.6	53.7		
K466R	Finger	5−270	2	0.087	20.9	21.1	21.3	151.5	146.1	163.4	73.9	61.3	68.6		
I472V	Finger	5−270	3	0.066	28.1	28.1	27.8	167.9	146.0	130.7	95.5	96.1	86.0		
P474L	Finger	33878	−3	0.109	25.4	25.9	26.1	80.1	106.7	153.5	58.4	77.7	85.5	69.86	75.83
I475L	Finger	5−270	2	−0.359	25.7	26.3	26.2	38.5	58.0	61.7	21.9	32.3	34.4		
R477K	Finger	5−270	2	0.303	22.5	22.5	22.7	85.4	74.4	96.1	35.9	36.3	46.9		
A485R	Finger	Kod−RI	−1	−2.007	14.8	15.3	14.9	30.9	31.4	17.9	28.4	13.2	7.5		
N491S	Finger	Kod−RS	1	0.376	10.6	10.5	10.1	48.8	35.1	37.0	33.6	29.7	31.3		
S493G	Finger	33878	0	1.666	13.2	13.2	12.7	0.0	0.7	0.0	0.0	0.6	0.0	47.71	74.31
E520Q	Palm	5−270	2	−0.031	15.2	14.9	14.8	88.1	79.7	70.6	50.6	44.6	39.5		
T523E	Palm	5−270	−1	−0.277	14.5	14.3	14.3	55.8	75.0	77.7	39.7	43.1	44.6		
M524T	Palm	5−270	−1	2.050	17.0	17.0	16.9	49.7	65.3	68.7	25.7	46.5	48.9		
K527R	Palm	5−270	2	−0.239	19.2	19.1	18.9	123.8	121.5	148.2	60.4	51.0	62.2		
Y533F	Palm	5−270	3	0.284	24.1	24.3	24.1	54.8	34.7	51.4	25.6	17.3	25.7		
I538L	Palm	5−270	2	−0.088	15.7	16.0	16.1	0.0	0.0	0.0	0.0	0.0	0.0		
S540A	Palm	5−270	1	−0.331	9.3	9.7	9.6	0.5	0.0	0.4	0.4	0.0	0.4		
T548P	Palm	33878	−1	6.284	19.7	19.8	20.0	3.6	0.5	3.2	2.6	0.4	2.3	25.85	47.91
P550H	Palm	33878	−2	1.468	25.1	25.1	25.8	91.1	87.2	169.9	66.4	63.6	92.9	60.16	68.26
M562K	Palm	5−270	−1	0.480	21.5	21.5	20.9	100.8	107.5	104.6	52.2	52.4	51.0		
K566D	Palm	5−270	−1	0.524	22.4	22.3	22.0	168.5	108.4	105.6	82.2	76.0	73.9		
A575L	Palm	5−270	−1	−1.199	17.7	17.6	17.4	25.4	84.7	77.9	23.4	47.2	43.4		
G602D	Thumb	5−270	−1	3.856	23.4	24.6	24.2	26.7	74.6	71.0	32.9	52.2	49.7		
R606G	Thumb	Kod−RSGA	−2	2.597	11.0	11.1	11.1	94.9	1.9	1.1	39.8	2.3	1.4		
D615G	Thumb	17349	−1	1.425	21.9	21.4	21.6	17.9	26.1	22.7	12.5	18.3	28.0	11.66	31.51
K672R	Thumb	5−270	2	−1.366	35.3	33.5	34.1	178.3	184.2	229.5	87.0	77.3	96.4		
P717S	Thumb	17349	−1	3.514	35.8	34.3	34.4	17.1	12.4	7.3	12.5	9.0	6.2	99.04	90.40
T723A	Thumb	Kod−RSGA	0	−0.079	47.7	46.0	46.4	118.9	96.5	92.2	84.5	88.7	84.8		
A741P	Thumb	23590	−1	3.358	23.1	23.2	23.0	0.2	0.8	1.3	0.2	0.8	0.9	17.28	19.32
F749C	Thumb	17349	−2	3.387	28.9			60.5			30.2			69.81	73.12
K771T	Thumb	17349	−1											73.05	74.55

References

1. Maola, V. A. *et al.* Directed evolution of a highly efficient TNA polymerase achieved by homologous recombination. *Nature Catalysis* **7**, 1173-1185 (2024).
2. Liao, J.-Y., Bala, S., Ngor, A. K., Yik, E. J. & Chaput, J. C. P(V) Reagents for the Scalable Synthesis of Natural and Modified Nucleoside Triphosphates. *J. Am. Chem. Soc.* **141**, 13286-13289 (2019).
3. Bala, S. *et al.* Synthesis of 2'-Deoxy-alpha-l-threofuranosyl Nucleoside Triphosphates. *J. Org. Chem.* **83**, 8840-8850 (2018).

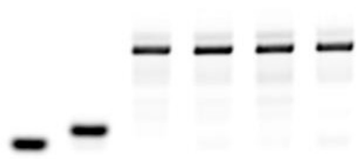

Uncropped gel for Supplementary Figure 8
