# Supplementary material for: Directed evolution of a TNA polymerase identifies independent paths to fidelity and catalysis
Source: Nat Commun. 2025 Dec 19;17:925. doi: 10.1038/s41467-025-67652-1 (PMC12830623; doi:10.1038/s41467-025-67652-1)
Supplement: Supplementary file 3 — Reporting Summary [file 41467_2025_67652_MOESM3_ESM.pdf]

nature portfolio

Corresponding author(s): John C. Chaput, Nicholas Chim

Last updated by author(s): Dec 1, 2025

Reporting Summary

Nature Portfolio wishes to improve the reproducibility of the work that we publish. This form provides structure for consistency and transparency in reporting. For further information on Nature Portfolio policies, see our Editorial Policies and the Editorial Policy Checklist.

Statistics

For all statistical analyses, confirm that the following items are present in the figure legend, table legend, main text, or Methods section.

n/a	Confirmed	
☐	☒	The exact sample size (*n*) for each experimental group/condition, given as a discrete number and unit of measurement
☐	☒	A statement on whether measurements were taken from distinct samples or whether the same sample was measured repeatedly
☒	☐	The statistical test(s) used AND whether they are one- or two-sided<br>*Only common tests should be described solely by name; describe more complex techniques in the Methods section.*
☒	☐	A description of all covariates tested
☒	☐	A description of any assumptions or corrections, such as tests of normality and adjustment for multiple comparisons
☐	☒	A full description of the statistical parameters including central tendency (e.g. means) or other basic estimates (e.g. regression coefficient) AND variation (e.g. standard deviation) or associated estimates of uncertainty (e.g. confidence intervals)
☒	☐	For null hypothesis testing, the test statistic (e.g. *F*, *t*, *r*) with confidence intervals, effect sizes, degrees of freedom and *P* value noted<br>*Give P values as exact values whenever suitable.*
☒	☐	For Bayesian analysis, information on the choice of priors and Markov chain Monte Carlo settings
☒	☐	For hierarchical and complex designs, identification of the appropriate level for tests and full reporting of outcomes
☒	☐	Estimates of effect sizes (e.g. Cohen's *d*, Pearson's *r*), indicating how they were calculated

Our web collection on statistics for biologists contains articles on many of the points above.

Software and code

Policy information about availability of computer code

Data collection	PAGE gels were imaged using Image Studio Lite v5.2 (LI-COR). Kinetics data were collected using a Synergy Neo2 plate reader with the Gen5 v3.14.03 software (BioTek). Progress of 2′-deoxy-α-L-threofuranosyl thymidine-3′-triphosphate (dtTTP) synthesis was monitored using a Bruker Avance NEO 400 NMR, a Waters (Micromass) LCT mass spectrometer, and a Thermo Scientific Dionex Ultimate 3000 HPLC system. The X-ray diffraction datasets were acquired at the Advanced Light Source (Beamlines 5.0.1 & 8.2.2), the Stanford Radiation Lightsource (Beamline 12-2), and the National Synchrotron Light Source II (Beamline AMX). AlphaFold3 structural predictions were performed on the online server provided by Google Deep Mind on 16 Aug 2024.
Data analysis	Fidelity experiments were analyzed by Sanger sequencing. DNA sequences for fidelity were analyzed using CLC Main Workbench 23.0.5 (Qiagen). Kinetics data were processed using Microsoft Excel v16.66. NMR spectra were analyzed using Top Spin 4.3. Mass spectrometry data were analyzed using Waters MassLynx 4.0. HPLC traces were analyzed using Chromeleon 7.2 SR4. The X-ray diffraction datasets were processed using XDS version:Jun 30 2023 or iMosflm v7.4.0. The initial models were determined using Phenix phaser v1.20.1-4487-000 and the final models were determined using iterative rounds of Coot v0.9.8.92 and Phenix refine v1.20.1-4487-000 and validated using Phenix Molprobity v1.20.1-4487-000. All structures were visualized using PyMol v2.4.0 and empirically determined structures were analyzed using web-based software, FreeSASA, FoldX, Web 3DNA 2.0, and PyVol 1.7.6, a PyMol plugin.

For manuscripts utilizing custom algorithms or software that are central to the research but not yet described in published literature, software must be made available to editors and reviewers. We strongly encourage code deposition in a community repository (e.g. GitHub). See the Nature Portfolio guidelines for submitting code & software for further information.

Data

Policy information about availability of data

All manuscripts must include a data availability statement. This statement should provide the following information, where applicable:

- Accession codes, unique identifiers, or web links for publicly available datasets
- A description of any restrictions on data availability
- For clinical datasets or third party data, please ensure that the statement adheres to our policy

Atomic coordinates are available through the Protein Data Bank (PDB) under the accession codes: 9OAT, 9OAU, 9OAV, 9OAW, 9OAX, 9OAY. The PDB also contains all other structures presented in this work. Furthermore, the authors declare that the data supporting the findings of this study are available within the article and its supplementary information files.

Human research participants

Policy information about studies involving human research participants and Sex and Gender in Research.

Reporting on sex and gender	not applicable
Population characteristics	not applicable
Recruitment	not applicable
Ethics oversight	not applicable

Note that full information on the approval of the study protocol must also be provided in the manuscript.

Field-specific reporting

Please select the one below that is the best fit for your research. If you are not sure, read the appropriate sections before making your selection.

[x] Life sciences [ ] Behavioural & social sciences [ ] Ecological, evolutionary & environmental sciences

For a reference copy of the document with all sections, see nature.com/documents/nr-reporting-summary-flat.pdf

Life sciences study design

All studies must disclose on these points even when the disclosure is negative.

Sample size	Kinetics: n=2 All time points were prepared and measured from three reaction replicates from a single master mix. Sample size was determined based on previous kinetics experiments in the field.<br>Fidelity: n = 20. Sanger sequencing was performed on 20 colonies from aggregate fidelity experiments, resulting in an error rate based on 1000 nts. Sample size was determined based on similar studies in aggregate fidelity experiments performed in the field.
Data exclusions	No data were excluded.
Replication	Kinetics: Absolute rates can vary based on sample preparation but the trends of catalytic rates among TNA polymerases are consistent. Rates are calculated from 2 replicates. All attempts at replication were successful.<br>All results shown in gel scans have been performed in, at least, duplicate and all attempts were successful.
Randomization	The experiments performed in this study require a rational approach for activity comparison and thus, randomization is not applicable to our experimental setup.
Blinding	The experiments performed in this study require a rational design. Therefore, blinding is not applicable to any biochemical assays and structural calculations performed in this study.

Reporting for specific materials, systems and methods

We require information from authors about some types of materials, experimental systems and methods used in many studies. Here, indicate whether each material, system or method listed is relevant to your study. If you are not sure if a list item applies to your research, read the appropriate section before selecting a response.

Materials & experimental systems

n/a	Involved in the study
☒	☐ Antibodies
☒	☐ Eukaryotic cell lines
☒	☐ Palaeontology and archaeology
☒	☐ Animals and other organisms
☒	☐ Clinical data
☒	☐ Dual use research of concern

Methods

n/a	Involved in the study
☒	☐ ChIP-seq
☒	☐ Flow cytometry
☒	☐ MRI-based neuroimaging
